# Structural adaptations of octaheme nitrite reductases from haloalkaliphilic *Thioalkalivibrio* bacteria to alkaline pH and high salinity

**DOI:** 10.1371/journal.pone.0177392

**Published:** 2017-05-16

**Authors:** Anna Popinako, Mikhail Antonov, Alexey Tikhonov, Tamara Tikhonova, Vladimir Popov

**Affiliations:** 1 Bach Institute of Biochemistry, Research Center of Biotechnology of the Russian Academy of Sciences, Leninsky Prospekt. 33, bld. 2, Moscow, Russian Federation; 2 M. K.Ammosov North-Eastern Federal University, suite 312, Yakutsk, Republic of Sakha (Yakutia), Russian Federation; 3 National Research Centre "Kurchatov Institute", Akad. Kurchatova sqr., 1, Moscow, Russian Federation; Montana State University Bozeman, UNITED STATES

## Abstract

Bacteria *Tv*. *nitratireducens* and *Tv*. *paradoxus* from soda lakes grow optimally in sodium carbonate/NaCl brines at pH range from 9.5 to 10 and salinity from 0.5 to 1.5 M Na^+^. Octaheme nitrite reductases (ONRs) from haloalkaliphilic bacteria of genus *Thioalkalivibrio* are stable and active in a wide range of pH (up to 11) and salinity (up to 1 M NaCl). To establish adaptation mechanisms of ONRs from haloalkaliphilic bacteria a comparative analysis of amino acid sequences and structures of ONRs from haloalkaliphilic bacteria and their homologues from non-halophilic neutrophilic bacteria was performed. The following adaptation strategies were observed: (1) strategies specific for halophilic and alkaliphilic proteins (an increase in the number of aspartate and glutamate residues and a decrease in the number of lysine residues on the protein surface), (2) strategies specific for halophilic proteins (an increase in the arginine content and a decrease in the number of hydrophobic residues on the solvent-accessible protein surface), (3) strategies specific for alkaliphilic proteins (an increase in the area of intersubunit hydrophobic contacts). Unique adaptation mechanism inherent in the ONRs from bacteria of genus *Thioalkalivibrio* was revealed (an increase in the core in the number of tryptophan and phenylalanine residues, and an increase in the number of small side chain residues, such as alanine and valine, in the core).

## Introduction

*Thioalkalivibrio* is a group of chemolithoautotrophic, obligately haloalkaliphilic *Gammaproteobacteria* within the *Ectothiorhodospiraceae* family. *Thioalkalivibrio* bacteria are found in soda lakes characterized by high pH [1] and moderate to high salinity, up to saturation. Optimal growth of closely related bacteria *Tv*. *nitratireducens* and *Tv*. *paradoxus* is observed in sodium carbonate/NaCl brines at pH from 9.5 to 10 and salinity from 0.5 to 1.5 M Na^+^. Octaheme nitrite reductases (ONRs) were isolated from periplasmic fraction of *Tv*. *nitratireducens* and *Tv*. *paradoxus* cell extract and were thoroughly characterized [2]. Both proteins exhibit high nitrite reductase activity in the six-electron reduction of nitrite to ammonia [3]. Apart from the nitrite reduction, ONRs can reduce hydroxylamine to ammonia, sulfite to sulfide, and hydrogen peroxide to water [3]. In solution, both enzymes exist as stable homohexamers [3]. The hexameric structure persists in pH range of 5–11. NaCl concentrations of up to 1 M stabilize the hexameric structure. The melting point determined by differential scanning calorimetry and circular dichroism increases from 71°C in the absence of salt to 86°C in the presence of 1 M NaCl [4].

**Fig 1:**
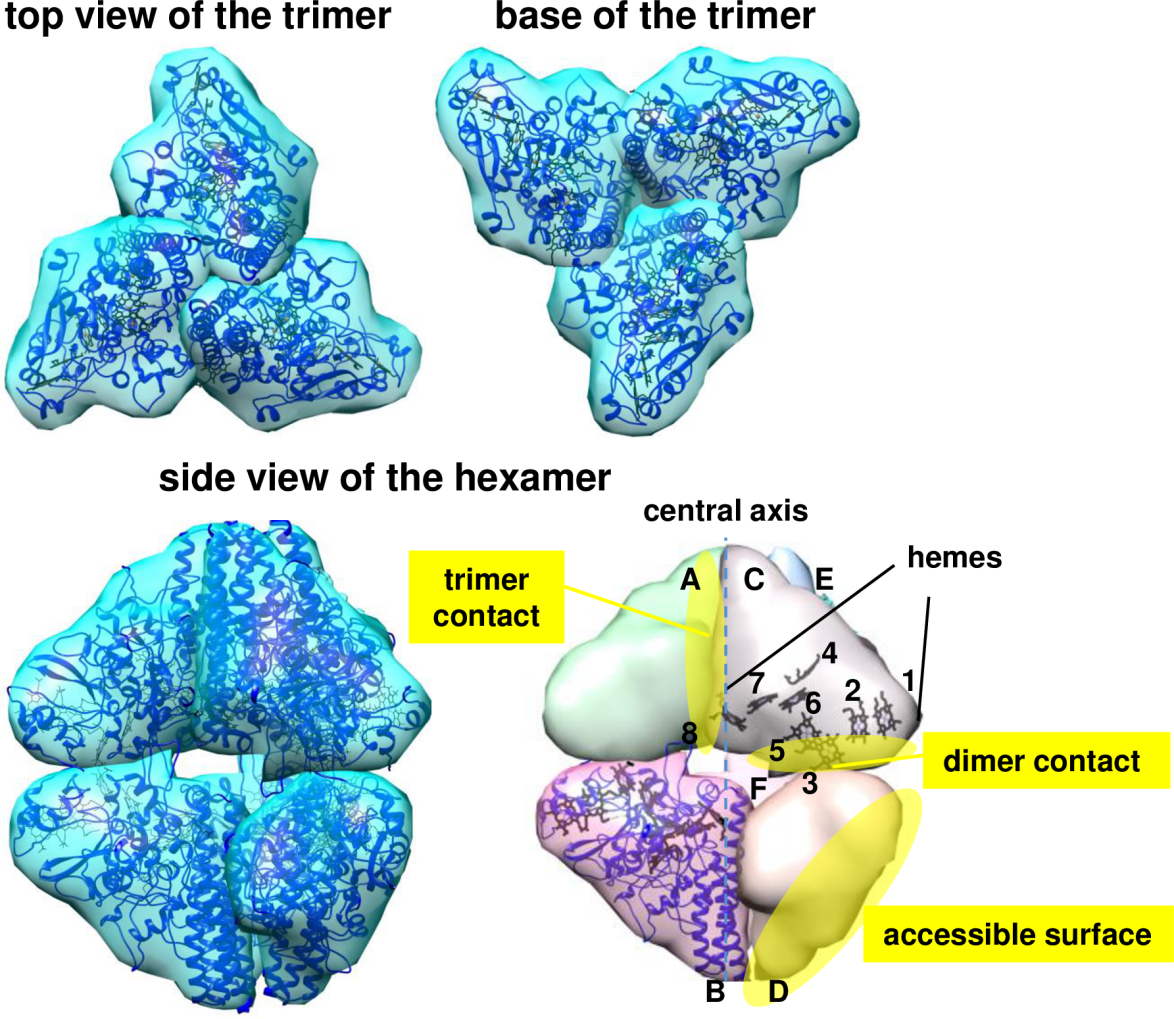
Shape of ONR from *Tv*. *nitratireducens* [5] (multiscale models of macromolecular assembly were generated with USCF Chimera). The figure shows the trimer (top view and view at the base) and the hexamer (side view). Subunits in the hexamer are designated by letters. Central axis is shown as a dotted line. Intersubunit contacts (trimers and dimers) and solvent-accessible protein surface are shown in yellow, hemes of the C subunit are numbered. Secondary structure of the B-subunit (cartoon representation) is shown in blue.

Structures of ONRs from *Tv*. *nitratireducens* (hereafter TvNiR) and *Tv*. *paradoxus* (TvPaR) were determined by X-ray crystallography at 1.5 Å and 1.9 Å resolution, respectively [3,5]. It was shown [5] that the TvNiR hexamer has a bipyramidal shape and is formed by a dimer of trimers with a buried surface area of 4850 Å^2^ per monomer [5]. The TvNiR subunits contain seven bis-histidine-coordinated c-type hemes (hemes 1–3 and 5–8) and one heme (heme 4, catalytic) coordinated by lysine through the amino group and a water molecule at the proximal and distal positions, respectively (Fig 1).

ONR homologue genes (sequence identity > 40%) were found in genomes of haloalkaliphilic bacteria (haONRs), as well as bacteria that grow best under neutral pH and at low salt concentrations (nnONRs). The ONR homologues share a set of common structural features [3]: 1) seven CXXCH and one CXXCK heme-binding motifs; 2) active site residues: tyrosine, arginine, cysteine. These common structural features allow to define a new family (ONRs).

The objective of this study was to analyze mechanisms of structural halo and alkali-adaptation in octaheme nitrite reductases with high stability under alkaline pH and high salinity.

Investigation of the mechanisms of protein adaptation are based mainly on a comparative analysis of amino acid sequences and three-dimensional structures of proteins from extremophilic bacteria and their analogues from bacteria living in non-extreme environments [6–10].

At the level of amino acid composition, adaptive strategy of halophilic proteins is based on an increase in the number of acidic amino acid residues (aspartate and glutamate) [9], a decrease in lysine content, a decrease in the number of hydrophobic residues with simultaneous replacement of residues containing bulky hydrophobic side groups (such as phenylalanine, methionine, leucine, and isoleucine) with small hydrophobic side chain residues (such as alanine, valine, and glycine) [9].

The adjustments in the amino acid composition of proteins from haloalkaliphilic bacteria are accompanied by unique changes in their three-dimensional structures [1,9,11–14].

One of the most common mechanisms of structural halo-adaptation relies on increased number of acidic residues on the solvent-accessible protein surface (+DE strategy) [7]. Clusters of negatively charged residues on the protein surface bind hydrated cations, thus preserving the hydration shell of the protein molecule and decreasing surface hydrophobicity. A negative surface charge results in electrostatic repulsion between protein molecules, thus preventing protein aggregation under high-salinity conditions [7,15,16]. This mechanism is universal and ensures, apart from halotolerance, high thermal stability of proteins. Adaptation of alkaliphilic proteins follows a similar mechanism [17]: the increased number of negatively charged amino acid residues results at lower pH values in the surface hydration layer of the protein.

Another mechanism of halophilic protein adaptation is based on a decrease in the number of surface lysine residues (-K strategy). The long hydrocarbon side chain of lysine located on the surface and exposed to the solvent substantially increases hydrophobicity of the surface [1,9,18]. Thus, lysine promotes protein aggregation under high salinity. In halophilic proteins, surface lysine residues are often replaced with more hydrophilic arginine (-K +R strategy) [6,19,20].

The decrease in surface lysine might be more important for stabilizing proteins in alkaline media, because lysine loses its positive charge under high pH [9] and its interaction with negative charged residues disappears. In alkaliphilic proteins unfavorable lysine-aspartate ion pairs are replaced by a more stable arginine-glutamate ion pairs (-DK+ER strategy) [9].

Another adaptation mechanism of halophilic proteins relies on a decrease in the number of hydrophobic residues on the protein surface (-YFIL strategy) [9]. These residues are replaced with glycine, alanine, and valine (+GAV strategy) [9], as well as with serine and threonine. This mechanism is associated with a decrease in hydrophobicity and an increase in the thickness of the hydration layer around the protein molecule. This strategy also leads to increased mobility of the polypeptide chain under high salinity [6,7].

Conversely, in the proteins from alkaliphilic organisms an increased number of hydrophobic residues in the intersubunit contacts and enhanced hydrophobic interactions in the interface are observed [17].

Another adaptation mechanism of halotolerant proteins involves an increase in the number of salt bridges between oppositely charged groups [9,21]. The abundance of salt bridges helps to maintain tertiary and quaternary protein structure under high salinity. In addition, the following possible adaptation mechanisms are suggested: a decrease in the solvent-accessible surface area [9] of the protein molecule and a decrease in the number of cysteine residues [19]. It remains unclear whether this -C strategy is specific for individual proteins or universal.

Adaptation mechanisms of haloalkaliphilic proteins were not studied extensively. We hypothesize that haloalkaliphilic organisms employ strategies common to halo- and alkaliphiles, but new adaptation mechanisms are also possible.

In this study three-dimensional models of ONRs from halophilic and non-halophilic neutrophilic microorganisms were obtained using homology modeling based on the structure of TvNiR from haloalkaliphilic bacterium *Tv*. *nitratireducens* [22]. A comparative structural analysis of these two subsets of ONRs was performed. Mechanisms of structural halo- and alkali-adaptation of ONRs were investigated in detail using two enzymes of this family as case studies: TvNiR and GsNiR from a neutrophilic non-halophilic bacterium *G*. *sulfurreducens* with 49% sequence identity.

## Materials and methods

### Bioinformatics analysis of amino acid composition of ONRs

The ONR sequences were obtained from BLAST [23] searches against the NCBI's nr database [24] (last searched November, 2016) with 500 sequences as a query and an e-value threshold of 1e-100. The search resulted in 64 ONR sequences with common structural features: presence of seven CXXCH and one CXXCK heme-binding motifs and presence of the active site residues (tyrosine, arginine, cysteine) (S1 Appendix). Sequences with higher e-values are only distantly related to this subfamily as they align to only 85% of the query and contain only 5 or 7 heme-binding sites. The ONR family is a very small group of proteins. Because all available sequences could be studied, the ONR family is a convenient object for investigation of specific structural adaptations. Phylogenetic tree was constructed with MEGA6 software [25] under default parameters (S1 Fig). We filtered sequences from this sample on the level of 90% identity, i.e. sequences that were identical by more than 90% to another sequence in the sample were removed. The sequences from thermophiles (4 sequences), from halophile (1 sequence), moderate salt-tolerant organisms, moderate thermophiles (8 sequences) and sequences from poorly explored organisms (25 sequences) were removed. Thus, the most representative proteins were selected. Multiple alignment of ONRs was generated with MUSCLE [26] and visualized using GeneDoc and Jalview [27]. Statistical analysis was performed with STATISTICA 7.0 software [28]. Comparison of amino acid compositions of ONRs from haloalkaliphilic and non-haloalkaliphilic organisms was carried out using the Mann-Whitney U-test [29]. The threshold for significance was set at *p* < 0.05.

### Homology modeling

All models were built based on the crystal structure of TvNiR (PDB ID 2OT4) [5]. Pairwise sequence alignment of the templates and the target sequence was performed with T-COFFEE software under default parameters [30]. MODELLER program [31] was used for homology modeling. The MODELLER protocol for homology modeling was selected in accordance with the tutorial (https://salilab.org/modeller/tutorial/advanced.html). Models produced by MODELLER were ranked according to moldpdf and DOPE scores to select model with the highest score. For example, the MODELLER objective function of the selected GsNiR hexamer model was 29073.4570. The selected energetically favorable models were used to investigate potential structural properties of ONRs from haloalkaliphilic and non-haloalkaliphilic bacteria. In order to refine the models, energy minimization was performed utilizing the Minimize subroutine of UCSF Chimera program under default parameters [32]. For standard residues Amber parameters were used [33]. The refined molecules were checked with WHAT IF [34]. Ramachandran plots were constructed utilizing PROCHECK [35]. Coordinates of all the models are available (S1 and S2 Files).

### Comparative analysis of ONRs

Interactive visualization and structural analysis of ONRs were performed using UCSF Chimera software [32]. SignalP 4.1 server (http://www.cbs.dtu.dk/services/SignalP/) was used for prediction of the presence and location of signal peptide cleavage sites in the ONR sequences [36]. Hydrophobic organization of interacting monomers in the ONR complexes and hydrophobic surfaces between monomers were analyzed using Platinum software [37]. Different types of contacts in ONR hexamers were calculated with CCP4 suite [38] and Protein Interactions Calculator (PIC) (http://pic.mbu.iisc.ernet.in/) [39]. Hydrophobic cores of ONRs were detected with CluD program (http://mouse.belozersky.msu.ru/npidb/cgi-bin/hftri.pl) [40]. Bioinformatics analysis of subfamily-specific positions responsible for halo- and alkali-adaptation within the ONR family was performed using Zebra (http://biokinet.belozersky.msu.ru/zebra) [41]. Two subfamilies were determined by Zebra which correspond to the group of non-haloalkaliphilic proteins (nnONRs) and the group of haloalkaliphilic proteins (haONRs). The subfamily-specific conservative positions for halo- and alkali-proteins were also determined with Zebra. Electrostatic potential was computed according to Coulomb's law using Chimera software package, thresholds ±10 kT. Chimera Coulombic Surface Coloring scheme was applied. Analysis of hydrophobic organization of interacting monomers in ONR complexes and verification of the refined ONR models was performed using Lomonosov supercomputer of the Supercomputing Center at the Moscow State University [42] and Arian Kuzmin supercomputer at the North-Eastern Federal University in Yakutsk (Russia).

## Results and discussion

### Comparative analysis of amino acid composition and primary structures of octaheme nitrite reductases

Homology searches for TvNiR using BLAST revealed 64 sequences with sequence identity of more than 40%, including 4 sequences from haloalkaliphiles, 4 sequences from thermophiles (*Caldimicrobium thiodismutans*, *Calditerrivibrio nitroreducens*, *Thermodesulfovibrio sp*. *RBG_19FT_COMBO_42_12*, *Candidatus Schekmanbacteria bacterium GWA2_38_9*), 1 sequence from halophile (*Desulfuromonas sp*. *TF*), 8 sequences from moderate salt-tolerant organisms, or moderate thermophiles, 22 sequences from non-halophilic neutrophiles and 25 sequences from poorly explored organisms. High similarity of the selected sequences and features specific for ONRs [3,43] indicated that these proteins belong to the ONR family. The number of residues in the multiple alignment of ONRs varied from 530 (in *Desulfuromonadales bacterium GWD2 54 10)* to 575 (in *Propionivibrio dicarboxylicus)*. The exception was a protein from *Mucispirillum schaedleri* which contained 619 residues due to additional loops 20–70.

**Fig 2:**
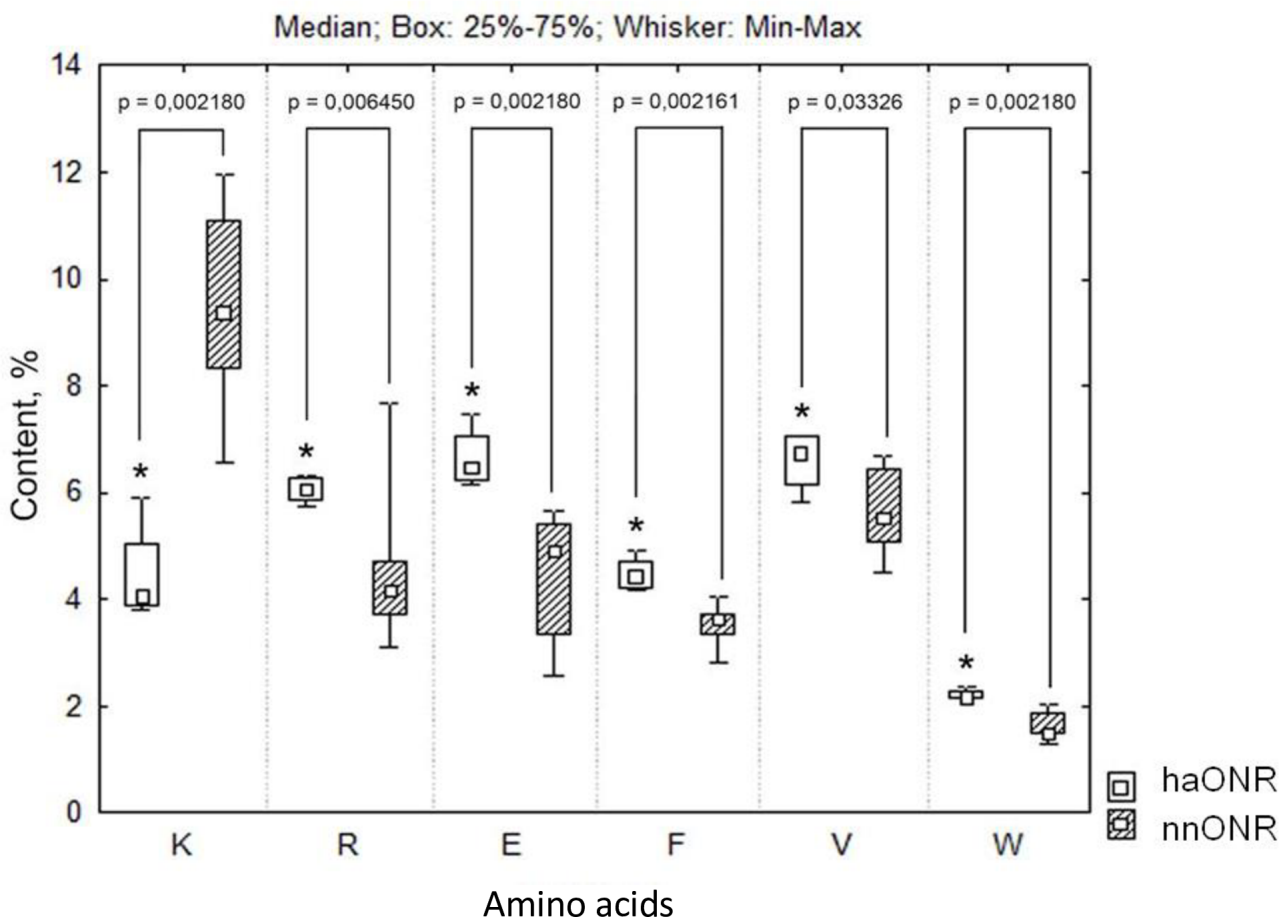
Comparative analysis of the amino acid composition of ONRs (amino acid residues are indicated in single letter code). *—*p* < 0.05 for comparison with nnONR.

Statistical analysis of the amino acid composition of the representative sample of halophilic ONRs (haONRs) (4 sequences) and non-halophilic neutrophilic ONRs (nnONRs) (22 sequences) showed a statistically significant (*p* < 0.05) decrease in the content of K residues and an increase in the content of R, E, F, V, and W residues in haONRs compared to nnONRs (Fig 2, S1 Table). For example, the median value for the content of E residues in haONRs is 24% higher compared to nnONRs; for R it is 31% higher. The K content in haONRs is 56% lower than in nnONRs, which is characteristic of the–K,+E adaptation strategies observed in both halophilic and alkaliphilic organisms and for the +R halo-adaptation strategy [9]. A change in the number of charged residues leads to a shift of pI for haloalkaliphilic ONRs by three units to the acidic range.

In the ONR family, a decrease in the percentage of bulky side chain amino acids (-YILF) with a simultaneous increase in the number of small side chain residues (+AGV) typical of protein halo-adaptation [6] is solely due to an increase in the V content in haloalkaliphilic proteins compared to proteins from non-haloalkaliphilic bacteria. In the ONR family, the total content of bulky side chain amino acids does not decrease. However, redistribution of these residues in the structure is observed (see section “Changes in core organization of ONRs associated with halo and alkali-adaptation”). Besides, an increase in the percentage of aromatic amino acids, which is not typical of halo-adaptation, is observed (the median value for phenylalanine in haONR is 17% higher compared to nnONR; for tryptophan, it is 31% higher; Fig 2). Usually, additional phenylalanine and tryptophan residues in haONRs are located in the vicinity of or inside the heme-binding motifs (S2 Table). Additional F/W are typical of the CFDCH motif of heme 1 in haONRs (S2 Table), the CFMCH motif of heme 5 in haONRs, the FNSF motif in the vicinity of heme 3, the QRRF motif in the vicinity of heme 4, the FAR motif in the vicinity of heme 5, the FQR, FRA, FDY, and FFW motifs in the vicinity of heme 6, the FWG motif in the vicinity of heme 7, and the EFWL motif in the vicinity of heme 8 in haONRs (S2 Table). Therefore, at the amino acid composition level, the mechanism of halo- and alkali-adaptation in haONRs combines protein adaptation strategies for alkaline and hypersaline environments (-K,+ER,+V strategies) and has specific features associated with changes in the percentage of aromatic amino acids (+F, +W).

### Comparative structural and bioinformatics analysis of haONRs and nnONRs

Apart from changes in the amino acid composition of haONRs compared to nnONRs, structural adaptation associated with distribution of different amino acid residues in the structure plays an important role. The most crucial structural locations are solvent-accessible surface, intersubunit contacts, and protein core. Three-dimensional models of ONRs from halophilic (S1 File) and non-halophilic neutrophilic microorganisms (S2 File) were obtained using homology modeling based on the structure of TvNiR from haloalkaliphilic bacterium *Tv*. *nitratireducens* [22]. A comparative structural analysis of these two subsets of ONRs was performed based on subfamily-specific positions identified using Zebra [41] with an emphasis on the two enzymes as case studies: TvNiR and GsNiR (49% sequence identity) from a neutrophilic non-halophilic bacterium *G*. *sulfurreducens* (Table 1, S2 and S3 Tables, S3 File).

**Table 1:** ONR structural analysis (hexameric state).

	Composition of solvent-accessible area of ONRs, surface occupied by residue in solvent-accessible area,%					Number of hydrophobic interactions in ONRs*	Composition of hydrophobic cores of ONRs number of atoms in core for residue,%								
	Hydrophobic residues*	K	R*	D*	E*		A	V	W*	F*	L	K*	I	M	H*
**TvNiR**	**19**	**7**	**12.3**	**12**	**15**	**102**	**4.1**	**8.9**	**7.8**	**14.8**	**10**	**4.9**	**4.6**	**5.8**	**10.4**
**Median, lower and upper quartile values (Me [Q1; Q3]) in haONRs**	**19.5** **[19;20.3]**	**7.6** **[7.4; 9.2]**	**12.8** **[11.5; 13.7]**	**9.9** **[9; 10.9]**	**13.2** **[11.4; 15]**	**100** **[97.5; 104]**	**4** **[3.3; 4]**	**8.9** **[8; 9]**	**8.6** **[7.8; 9]**	**14.4** **[13.3;14.9]**	**9.3** **[8.3; 9.5]**	**5.2** **[4.8;5.4]**	**6.9** **[4.9; 7]**	**5.8** **[5; 6.1]**	**10.4** **[9.3; 10.6]**
**GsNiR**	**20**	**23.6**	**6.5**	**10.8**	**9.1**	**47**	**3.1**	**6.3**	**6**	**11.2**	**10.2**	**10.4**	**6.9**	**4.8**	**9.7**
**Median, lower and upper quartile values (Me [Q1; Q3]) in nnONRs**	**31** **[29;32.3]**	**11.1** **[10.3; 11.6]**	**5.9** **[5.5; 6.6]**	**6.3** **[5.4; 7.3]**	**5.6** **[4.1; 7.2]**	**56.5** **[48.8; 64]**	**3.3** **[3; 3.7]**	**7.2** **[6.9; 8.5]**	**6.1** **[8.3; 6.8]**	**12** **[11.2–12.2]**	**9.5** **[9.2; 10.3]**	**12** **[10.4; 13.7]**	**5.7** **[4.6; 4.8]**	**5.3** **[4.2; 6.3]**	**8.7** **[7.6; 9.2]**

*—p (probability corresponding to U-Mann-Whitney U-test) < 0.05 for comparison with nnONR

#### Halo- and alkali-adaptation of haONR through redistribution of amino acids on the solvent-accessible surface

**Fig 3:**
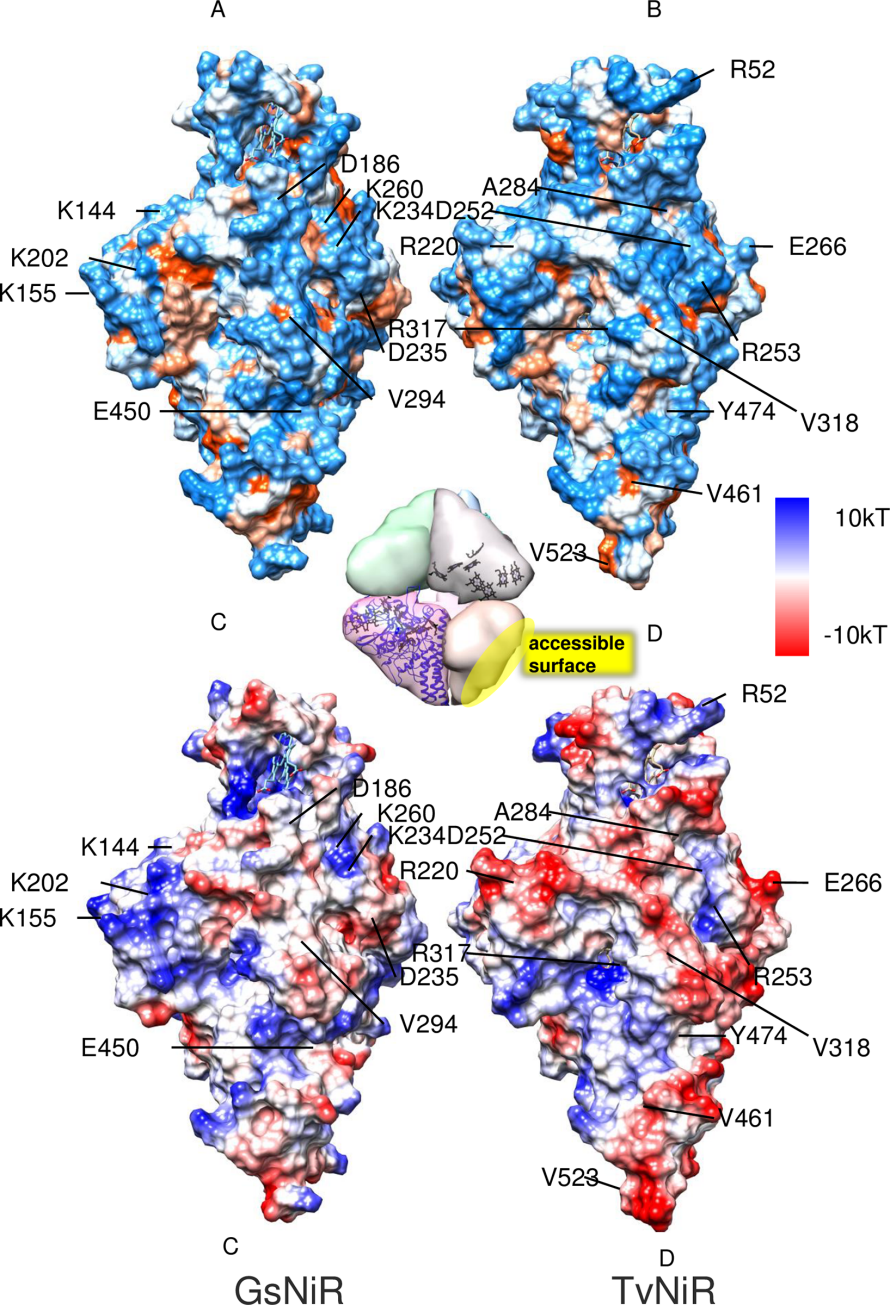
Solvent-accessible surfaces of GsNiR (A) and TvNiR (B), hydrophobic regions are highlighted in orange, hydrophilic in blue. Conserved substitutions on the solvent-accessible surface are shown by arrows. The inset shows localization of the solvent-accessible surface in the hexamer. Solvent-accessible surface of GsNiR (C) and TvNiR (D) with relative electrostatic surface potentials. The surface of TvNiR is charged negatively.

The stabilization of the hexameric structure of TvNiR under high-salinity conditions and at alkaline pH values is due to a decrease by about 15% in the total solvent-accessible surface area of TvNiR compared to GsNiR (Table 2); a similar mechanism was revealed in proteins from other halophilic organisms [9]. The surface of hydrophobic residues in the solvent-accessible area of the TvNiR hexamer is also smaller than that in GsNiR (Table 1, Fig 3).

**Table 2:** Complementarity of hydrophobic/hydrophilic properties in ONRs (Sll–hydrophobic-hydrophobic buried surface area in ONR intersubunit contacts, Shh–hydrophilic-hydrophilic buried surface area between subunits, Sburied–total area of contact between subunits (buried surface area), Stotal–total surface area, Sasa–surface accessible area).

Type of contact	Enzyme	Sll, Å^2^	Shh, Å^2^	Sburied, Å^2^	Stotal, Å^2^	Sasa, Å^2^
Dimer	GsNiR TvNiR	16.0 121.8	824.5 526.1	1273.3 902.3	22930.5 22453.1	21657.3 21549.8
Trimer	GsNiR TvNiR	69.8 203.0	803.6 500.9	1371.1 1287.6	22930.5 22453.1	21559.4 21165.5
Hexamer	GsNiR TvNiR	466.4 1583.5	7295.2 4583.9	12046.5 10432.4	100474.7 84396.0	100474.7 84396.0

The comparative structural analysis of TvNiR and GsNiR revealed a 70.3% decrease in the area of lysines on the solvent-accessible surface of TvNiR (Table 1). The lower lysine content in TvNiR compared to GsNiR makes the main contribution to a decrease in the hydrophobic solvent-accessible surface area of the TvNiR (Table 1). About 45% of surface lysines in GsNiR are replaced in the TvNiR structure with nonpolar residues with low hydrophobicity (S4 Table). Nonpolar substitutions lead to an additional decrease in the hydrophobicity of the solvent-accessible surface of TvNiR because these residues are buried in the surface interface. The corresponding K residues on the surface of GsNiR form a raised surface. The remaining 55% of surface lysines of GsNiR are replaced with polar and charged amino acids in the TvNiR structure (S4 Table). A comparative structural analysis of TvNiR and GsNiR revealed a 10% and 39% increase in the number of D and E residues (Table 1), respectively, on the solvent-accessible surface of TvNiR, making the surface of TvNiR molecule more electronegative than that of GsNiR. The negative charge of the molecule contributes to stabilization of TvNiR in hypersaline environments due to weakened aggregation as a result of repulsion between solvent-accessible surfaces of the hexamers bearing the same charge. At alkaline pH resulting in a deficit of protons, the overall negative charge of the TvNiR attracts protons required for catalysis. Besides, the percentage of the number of arginine residues on the solvent-accessible surface of TvNiR is 47% higher compared to GsNiR (Table 1, Fig 3) which is characteristic of the classic adaptation strategy (+R) for halo- and alkaliphiles [9].

Bioinformatics analysis of positions specific for TvNiR and GsNiR, as well as the haONR and nnONR subfamilies (S2 Table) and ONR structural analysis (Table 1, S2 Table, S1, S2 and S3 Files) revealed a number of conserved substitutions resulting in a decrease in the number of hydrophobic amino acids and K residues on the solvent-accessible surface (Table 1, S2 Table, for example, 155, 52, 284, 179, 220, 375 positions) and an increase in the number of surface E, R, D (Table 1), G, S, and T residues on the solvent-accessible surface (positions 155, 265, 179, 290 in S2 Table). Thus, the comparison of haONR subset against nnONR subset as well as the pairwise comparison of TvNiR and GsNiR revealed the same main strategies specific for halophilic and alkaliphilic proteins (an increase in the number of glutamate and aspartate residues, a decrease in the number of lysine residues, hydrophobic residues and an increase in the arginine content on the protein surface, Table 1, S2 Table).

#### Changes in intersubunit contacts in ONRs associated with halo- and alkali-adaptation

As mentioned above, the characterized haONR representatives (TvNiR and TvPaR) exist in solution, and in crystals as homohexamers [5]. Stability of the hexamers increases with an increase in NaCl concentration in solution, which can be considered a result of protein adaptation to high-salinity environments [4].

**Fig 4:**
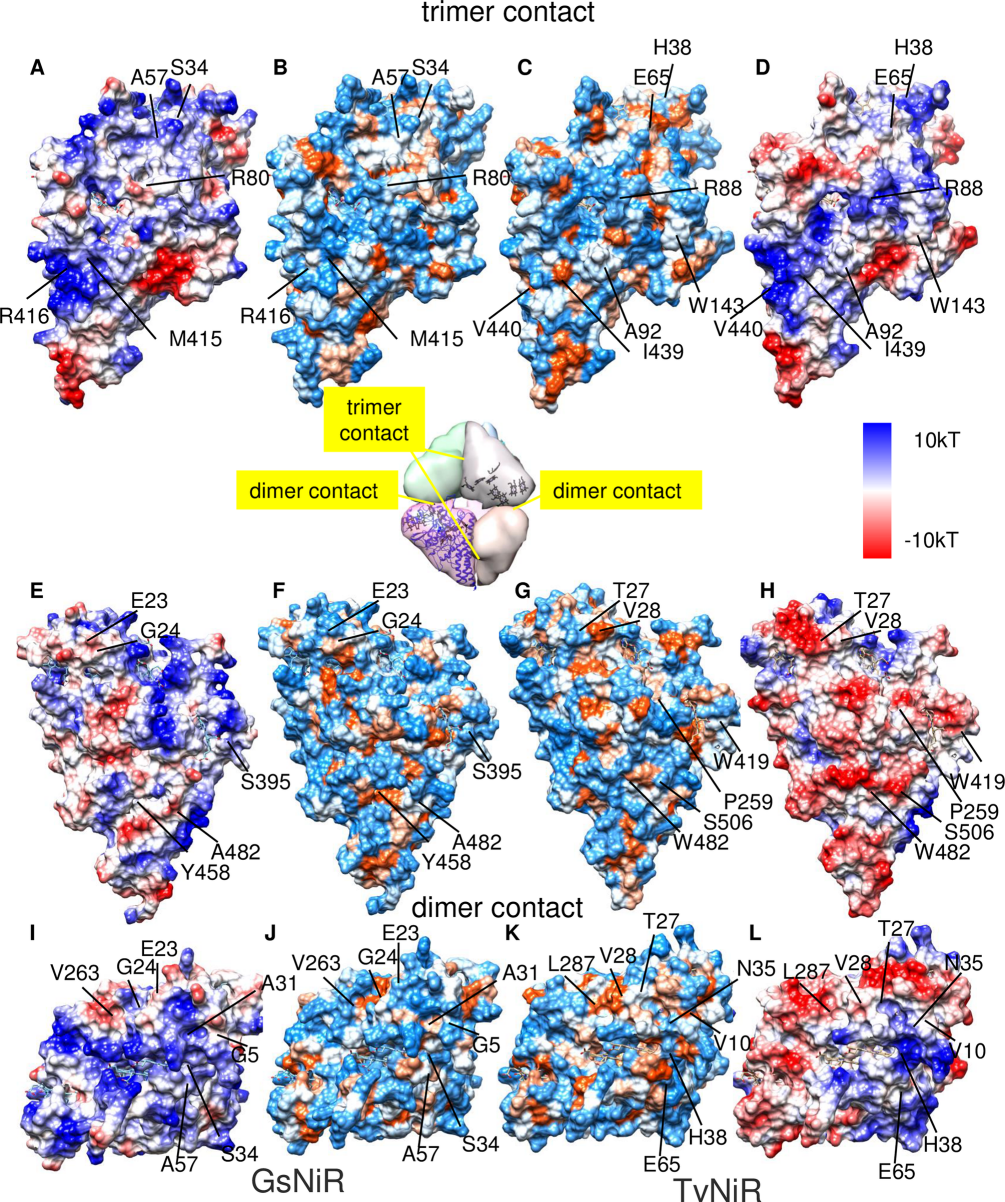
Intersubunit contacts in GsNiR (the trimer contact (A, B, E, F,) and the dimer contact (I, J,)) and TvNiR (the trimer contact (C, D, G, H) and the dimer contact (K, L)); hydrophobic regions are highlighted in red, hydrophilic are blue. The inset in the center of the figure shows localization of contacts in the hexamer. The intersubunit contacts in GsNiR and TvNiR are shown with relative electrostatic surface potentials on left (A, E, I) and right (D, H, L) respectively.

An analysis of intersubunit contacts demonstrated that the TvNiR structure is characterized by larger hydrophobic surface areas (Fig 4, Table 2) compared to GsNiR. The surface area of hydrophobic regions in the dimer and trimer contacts in TvNiR is 7.6 and 2.9 times larger, respectively, than that in GsNiR. Mutual arrangement of hydrophobic residues at distances shorter than 5 Å is energetically most favorable and can be considered as hydrophobic interaction [44]. The dimer contact region of TvNiR involves hydrophobic P9(A)−A32(B) and P9(B)−A32(A) interactions. Hydrophobic intersubunit interactions were not found in the dimer contact in the GsNiR structure.

According to calculation of intersubunit interactions, the number of hydrophobic interactions in the trimeric contact of TvNiR is 52% larger compared to GsNiR due to the following amino acid replacements: T103(GsNiR) with A111(TvNiR), A136(GsNiR) with T147(TvNiR), P157(GsNiR) with S175(TvNiR), E158(GsNiR) with Q176(TvNiR), S395(GsNiR) with W419(TvNiR), and A482(GsNiR) with S506(TvNiR) (S4 Table). Apart from classic hydrophobic interactions, the TvNiR trimer is stabilized by additional F140(A)−Y430(E), W143(A)−Y430(E), and W143(A)−Y434(E) stacking interactions, which are absent in GsNiR. The TvNiR trimer is also characterized by an increased number (by 53%) of T-stacking interactions compared to GsNiR (due to the presence of Y426(F)−H83(B), Y426(F)−R118(B), Y430(F)−R118(B), and Y434(F)−R88(B) interactions).

The tendency to increase in the number of hydrophobic interactions is observed in the haONR subfamily as a whole (Table 1). Bioinformatics analysis of positions specific for the haONR and nnONR subfamilies and ONR structures comparison (S2 Table) showed that residues involved in hydrophobic interactions, as well as stacking and T-stacking interactions, are conserved among haONRs. This attests to the common mechanism of stabilization of the oligomeric structure for all haONRs. Therefore, in high-salinity environments and at alkaline pH values, an increase in the surface area of intersubunit hydrophobic contacts, along with stacking and T-stacking interactions, makes a considerable contribution to stabilization of the oligomeric structure of haONR. This is due to weakened intersubunit electrostatic interactions and strengthened hydrophobic interactions under conditions of low water activity [45].

Apart from hydrophobic interactions, intersubunit contacts in haONR are stabilized through the hydrophilic-hydrophilic contact area (Table 2), which is much larger than the hydrophobic contact area. Redistribution of intersubunit hydrogen bonds plays a crucial role in stabilization of haONRs in haloalkaliphilic conditions. Intersubunit hydrogen bonds K8(A)−T27(B), V28(A)−N6(B), T33(A)−H38(B), and E65K(A)−K394(B) in the dimer contact and R88(A)−Y430(E), Y434(E) hydrogen bonds in the trimer contact (Fig 4) of TvNiR, conserved for haONRs (S2 Table, ONR structures comparison S1 and S2 Files), stabilize the TvNiR hexamer in the peripheral region in respect to the central symmetry axis of the protein (highlighted in the figure). To reduce the effect of high salinity, charged residues involved in the intersubunit contacts in TvNiR are shielded by hydrophobic amino acids (I439, V28, V440, *etc*.) conserved in haONRs (S2 Table, S1 and S2 Files). There are no such hydrogen bonds in the GsNiR structure. As long as there are no hydrophobic interactions and salt bridges in GsNiR’s dimer interface, it is probable that the GsNiR molecule is more stable as a trimer. Apart from the TvNiR, the GsNiR’s trimer is additionally stabilized along the central axis by the hydrogen bonds in the regions of the K81−Q93 and P362−H375 loops.

#### Changes in core organization of ONRs associated with halo- and alkali-adaptation

In addition to surface changes, which contribute to stability and prevent aggregation, adaptation of proteins to extreme environments is achieved through changes and redistribution of contacts and bonds within the protein globule.

**Fig 5:**
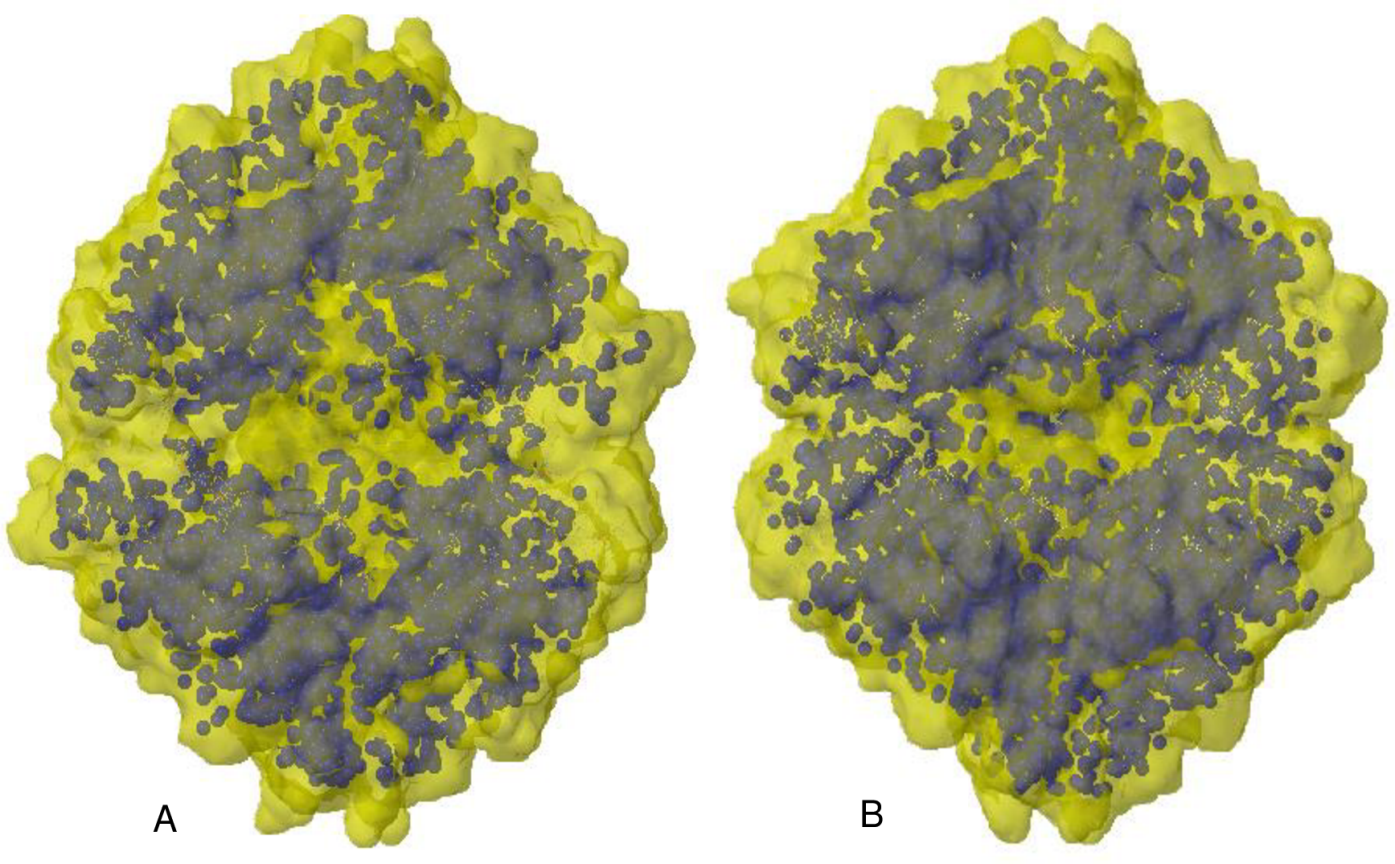
Hydrophobic core of GsNiR (A) and TvNiR (B). The hydrophobic core is highlighted in blue and presented as spheres. The protein surface is transparent and highlighted in yellow.

Comparative analysis of the structure and composition of hydrophobic cores of TvNiR and GsNiR (Fig 5, S3 Table) shows that the core in TvNiR has larger volume and is less shielded from the environment compared to the core in GsNiR. In the hydrophobic core of the TvNiR hexamer, content of conformationally more flexible hydrophobic residues with a relatively small side chain (A, V) is 28% higher, whereas percentage of large non-polar residues (I, L) is 15% lower compared to GsNiR (Table 1, S3 Table).

The lysine content in the core of TvNiR is also 53% lower than that in the core of GsNiR (Table 1, S2, S3 and S4 Tables). All these substitutions lead to an increase in the flexibility of the core of TvNiR.

On the other hand, the number of aromatic residues (F, W, H) in the core of TvNiR is 18% larger than that in GsNiR (Table 1, S3 Table). Interplanar distances between additional F/W residues (FNSF, QRRF, FAR, FQR, FRA, FFW, FWG, and EFWL motifs) and aromatic residues involved in heme protein environment correspond to stacking interactions [46]. Stacking interactions between aromatic residues are energetically more favorable under conditions of low water activity [47], resulting in additional stabilization of the core (S2 and S4 Tables).

The comparative analysis of the amino acid composition of the hydrophobic cores in haONR and nnONRs (Table 1) shows that the content of the conformationally flexible hydrophobic residues with a relatively small side chain (A, V) is on average by 19% higher, whereas percentage of the large non-polar residues (L) is 2% lower compared to nnONRs (S3 Table). The lysine content in the core of haONRs is also 57% lower than that in the core of nnONRs (Fig 2, S2, S3 and S4 Tables, S1 and S2 Files). The average number of aromatic residues (F, W) in the core of haONRs is 21% higher than that in nnONRs (S3 Table).

Therefore, bioinformatics analysis of positions specific for the haONR and nnONR subfamilies (Table 1, S2 Table) and ONR structures comparison suggests that adaptation of haONR at the core level relies on the balance between stability (through an increase in the number of stacking interactions between the aromatic residues, such as F, W, H, and Y, in the core) and conformational flexibility (through an increase in number of conformationally flexible hydrophobic residues, such as A, V, and M).

An additional W419 residue in all haONR hexamers is involved in hydrophobic interactions with the CLNCH heme-binding motif of heme 8 (S2 Table, S1 File). An increase in the hydrophobicity of the heme environment due to the involvement of additional aromatic residues (F and W) can also lead to a change in the redox potential of the hemes and, as a consequence, to a change in catalytic activity [48,49].

The heme environment in haONR (S2 Table, S1 File) involves also S-aromatic interactions between additional phenylalanine residues and nearest methionines (F16−M13 for heme 1, F75−M150 for heme 3, F229−M62 for heme 5). These interactions can prevent methionine oxidation by reactive oxygen species [50]. This is particularly important in case of alkali-adaptation because at high pH values reactive oxygen species can become more stable [51].

Therefore, classic strategies based on an increase in the percentage of small side chain residues (+AV) and a decrease in the percentage of large side chain residues (-L) and unique strategy based on increase in the percentage of aromatic residues (+FWH) take place in haONR at the core level. These results are consistent with data on an increase in the percentage of small hydrophobic residues in the hydrophobic core of archaeal dihydrofolate reductases, resulting in an increase in conformational flexibility and, consequently, in enzyme activity in hypersaline environments [6].

## Conclusions

Octaheme nitrite reductases (ONRs) from haloalkaliphilic bacteria use several strategies for adaptation to alkaline pH and high salinity, including those described earlier for halo- and alkaliphilic proteins, and unique strategies which have not been discussed previously.

A comparison of the amino acid compositions of ONRs from haloalkaliphilic (haONRs) and neutrophilic non-halophilic (nnONRs) revealed adaptation strategies specific for both halophilic (+E, +V, -K, +R) and alkaliphilic (+E, -K) proteins. Besides, we found an increase in the percentage of tryptophan (+W) and phenylalanine (+F) in haONRs, which has not been described earlier for haloalkaliphiles.

The detailed structural analysis was performed for two representatives of the ONR family from a haloalkaliphilic bacterium *Thioalkalivibrio nitratireducens* (TvNiR) and a non-halophilic neutrophilic bacterium *Geobacter sulfurreducens* (GsNiR). The generalities derived from this pairwise comparison were verified by extending the structural analysis using two subgroups–haONRs and nnONRs.

A comparative structural analysis of TvNiR and GsNiR in terms of hydrophobicity and hydrophilicity showed that changes in the amino acid composition are accompanied by redistribution of residues in the structure, resulting in stabilization and maintenance of functional properties of haONR under high-salinity and alkaline conditions. For instance, an increased number of charged residues (D, E, R) is observed on the solvent-accessible surface of the haONR hexamer. Additional D and E residues form negatively charged networks on the protein surface. This adaptation strategy ensures attraction of protons, which are deficient at alkaline pH, and weakens aggregation of protein molecules under high-salinity conditions. Weakened aggregation in hypersaline environments is also facilitated by a considerable decrease in the hydrophobic solvent-accessible surface area of the haONR hexamer compared to nnONR.

In extreme conditions, the TvNiR hexamer is stabilized due to a considerable increase in the surface area of hydrophobic-hydrophobic contacts between the subunits of the TvNiR hexamer compared to GsNiR. Apart from hydrophobic interactions, additional salt bridges in the peripheral region with respect to the central axis of the hexamer contribute to stability of the hexameric structure of TvNiR in alkaline high-salinity environments. Charged residues of TvNiR, which are involved in salt bridges, are shielded from environmental salt ions by hydrophobic residues.

The strategy based on an increase in the percentage of small hydrophobic residues (+A,+V) and a decrease in the percentage of large hydrophobic residues (-K, -L), which is typical of halophilic proteins, takes place at the hydrophobic core level in haONRs and results in an increase in conformational flexibility and, consequently, in activity of the enzyme in hypersaline environments.

A unique strategy associated with an increase in the percentage of aromatic residues (+W, +F) in the amino acid composition was discovered for haONRs. Intramolecular interactions between the additional aromatic residues conserved for haONRs (+W, +F) stabilize the molecule and lead to an increase in hydrophobicity of the heme environment. An increase in hydrophobicity of the protein environment of the hemes can lead to changes in redox potentials of the latter and in catalytic activity of the enzyme. Besides, the heme environment in haONRs involves S-aromatic interactions between additional phenylalanine resides and nearest methionines. These interactions are particularly important for alkali-adaptation because, at high pH values, reactive oxygen species become more stable.

Therefore, adaptation of TvNiR to low water activity conditions relies on the balance between stability (through the +E,-K,+R and +D strategies on the protein surface, an increase in the number of aromatic residues (F, W, H in the core), a decrease in the hydrophobic solvent-accessible surface area, strengthening of hydrophobic-hydrophobic interactions between the subunits of the hexamer, an increase in the number of hydrogen bonds) and conformational flexibility (due to an increase in the percentage of conformationally flexible hydrophobic residues in the core (A, V) and a decrease in the number of large side chain residues (K, L) in the core).

The authors are grateful to T.N. Safonova, V.N. Novoseletsky, A.N. Diakonova, D.V. Dibrova, Naveen Kumar and I.A. Dem’yanenko for the help, discussions and assistance in editing of the manuscript.

## Supporting information

S1 Table Comparison of the amino acid compositions of ONRs. (DOC)

S2 Table Subfamily-specific amino acid residues and structural comparison of ONRs. (DOC)

S3 Table Comparison of hydrophobic cores of ONRs. (DOC)

S4 Table Amino acid substitutions between GsNiR and TvNiR. (DOC)

S1 Appendix Sequences of 64 ONRs. (DOC)

S1 Fig Phylogenetic analysis of ONRs. (DOCX)

S1 File The models of haONRs. (PDB)

S2 File The models of nnONRs. (PDB)

S3 File Comparative analysis of ONRs (amino acid residues are indicated in single letter code). *—p < 0.05 for comparison with nnONR.
(DOCX)

## Abbreviations

ONR: Octaheme Nitrite Reductase
haONR: ONR from haloalkaliphilic microorganisms
nnONR: ONR from non-halophilic neutrophilic microorganisms
GsNiR: nnONR from *Geobacter sulfurreducens*
TvNiR: haONR from *Thioalkalivibrio nitratireducens*
