# Supplementary material for: Structural adaptations of octaheme nitrite reductases from haloalkaliphilic Thioalkalivibrio bacteria to alkaline pH and high salinity
Source: PLoS One. 2017 May 16;12(5):e0177392. doi: 10.1371/journal.pone.0177392 (PMC5433712; doi:10.1371/journal.pone.0177392)
Supplement: S1 Table — (DOC) [file pone.0177392.s001.doc]

S1. Сomparison of the amino acid compositions of ONRs from haloalkaliphilic and non-haloalkaliphilic organisms was carried out using the Mann-Whitney U-test. Proteins from haloalkaliphilic organisms are highlighted in blue.

| protein|organism | Id,% | pI | K,% | R,% | E,% | F,% | V,% | W,% |
| --- | --- | --- | --- | --- | --- | --- | --- | --- |
| Q5F2I3|Tv. nitratireducens | 100 | 5.91 | 3.80 | 6.33 | 6.33 | 4.52 | 7.05 | 2.17 |
| E7EDQ7|Tv. paradoxus | 81 | 5.95 | 3.98 | 5.97 | 6.15 | 4.16 | 7.05 | 2.17 |
| Q1NQZ7|delta proteobacterium MLMS-1 | 54 | 5.63 | 4.18 | 6.18 | 7.45 | 4.91 | 5.82 | 2.36 |
| D6Z5C1|Desulfurivibrio alkaliphilus | 54 | 6.6 | 5.92 | 5.75 | 6.64 | 4.31 | 6.46 | 2.15 |
| A1ATC6|Pelobacter propionicus | 49 | 8.93 | 6.55 | 7.68 | 5.43 | 3.37 | 5.99 | 1.50 |
| E8WT73|Geobacter sp. (strain M18) | 49 | 8.96 | 11.36 | 4.10 | 5.21 | 3.54 | 6.52 | 1.30 |
| B5EIZ4|Geobacter bemidjiensis Bem | 48 | 9.03 | 11.52 | 3.90 | 5.20 | 3.53 | 5.39 | 1.49 |
| Q74G90|G. sulfurreducens | 49 | 9.0 | 10.51 | 4.69 | 5.25 | 3.19 | 4.88 | 1.69 |
| A5G8N0|Geobacter uraniireducens | 49 | 9.01 | 11.94 | 4.85 | 5.60 | 3.54 | 5.41 | 2.05 |
| B3E660|Geobacter lovleyi SZ | 48 | 9.15 | 11.09 | 3.70 | 4.99 | 3.33 | 6.28 | 1.48 |
| C6E3S2|Geobacter sp. (strain M21) | 47 | 8.95 | 11.34 | 4.09 | 5.39 | 3.90 | 5.58 | 1.49 |
| B9M3N3|Geobacter daltonii | 46 | 8.86 | 10.15 | 5.26 | 5.64 | 2.82 | 5.08 | 1.69 |
| R7C1Y6|Sutterella sp. CAG:397 | 45 | 9.14 | 9.44 | 4.81 | 3.70 | 3.89 | 6.48 | 1.48 |
| K1K0I9| Sutterella sp. KLE1602 | 43 | 8.58 | 9.29 | 4.46 | 4.46 | 3.90 | 5.58 | 1.49 |
| D9Y3D5|Burkholderiales bacterium | 42 | 8.17 | 7.29 | 3.10 | 2.91 | 3.64 | 4.92 | 2.00 |
| R6A2A8|Proteobacteria bacterium | 42 | 8.44 | 7.29 | 3.10 | 2.55 | 3.64 | 4.92 | 2.00 |
| S3BDI3|Sutterella wadsworthensis HGA0223 | 41 | 8.29 | 8.46 | 4.23 | 3.31 | 3.68 | 6.43 | 1.47 |
| E7H1H6|Sutterella wadsworthensis 3_1_45B | 41 | 8.96 | 8.35 | 4.27 | 3.34 | 3.71 | 6.68 | 1.48 |
| R5Q3C1|Sutterella wadsworthensis CAG:135 | 41 | 8.29 | 8.46 | 4.23 | 3.31 | 3.68 | 6.62 | 1.47 |
| H3KEV4|Sutterella parvirubra YIT 11816 | 40 | 7.85 | 8.72 | 4.08 | 5.57 | 3.34 | 5.38 | 1.86 |
| A0A1F9PNL8_9DELT|OGR30974.1 |Desulfuromonadales bacterium GWD2 54 10 | 44 | 8.96 | 11.32 | 3.77 | 4.71 | 4.15 | 5.66 | 1.32 |
| A0A1F9PS59  |OGR32206.1 |Desulfuromonadales bacterium GWD2 61 12 | 42 | 8.30 | 8.94 | 4.47 | 5.77 | 3.91 | 6.51 | 1.86 |
| SDI64766.1|Propionivibrio dicarboxylicus | 53 | 8.85 | 9.21 | 4.34 | 4.17 | 3.48 | 5.91 | 1.74 |
| A0A0A8WS53  |WP 041971764.1 |Geobacter sp. OR-1 | 44 | 9.00 | 10.9 | 5.0 | 5.45 | 3.0 | 5.0 | 1.50 |
| A0A0B5BCG8 |WP_039739996.1 |Geobacter pickeringii | 43 | 8.94 | 11.38 | 4.77 | 5.87 | 3.85 | 6.0 | 1.83 |
| A0A0C1QSS6 |WP 039647922.1|Geobacter soli | 45 | 8.65 | 10.13 | 4.69 | 5.81 | 3.38 | 5.44 | 1.69 |
| P(probability corresponding to U-Mann-Whitney U-test) | | | 0.002180 | 0.006450 | 0.002180 | 0.002161 | 0.03326 | 0.002180 |
