# Supplementary material for: Structural adaptations of octaheme nitrite reductases from haloalkaliphilic Thioalkalivibrio bacteria to alkaline pH and high salinity
Source: PLoS One. 2017 May 16;12(5):e0177392. doi: 10.1371/journal.pone.0177392 (PMC5433712; doi:10.1371/journal.pone.0177392)
Supplement: S2 Table — (DOC) [file pone.0177392.s002.doc]

S2. Subfamily-specific amino acid residues and structural comparison of ONRs. Residues belonging to different regions are color coded. Core amino acids within the subunit are highlighted in white. Accessible surface is highlighted in blue. Dimer contacts are highlighted in green. Trimer contacts are highlighted in yellow. The residue number (according to the TvNiR structure) is in the first column. The probability of occurrence of a particular residue in percent in columns 2 and 3 in parenthesis are based on the subset of ONRs. Comments concerning effect on the structure (generally for haONRs compared to nnONRs) are in the 4th column.

| **Residue** | **haONR** | **nnONR** | **Structural role** |
| --- | --- | --- | --- |
| 127H | H(100%) | Q(100%) | Core amino acids within subunit hydrogen bonds. 127H is included in DHF motif from local environment of heme 4 in haONRs |
| 368F | F(100%) | Y(94%) | Decrease in the number of core amino acids within subunit hydrogen bonds (8 for GsNiR, 5 for TvNiR). 368F is included in FWG motif from local environment of heme 7 in haONRs |
| 218F | F(100%) | V(61%), M(17%) | Core amino acids within subunit hydrophobic interaction. 218F is included in FAR motif from local environment of heme 4 in haONRs |
| 134Y | Y(100%) | Q(33%), F(44%) | Stacking interactions in haONRs, core amino acids within subunit hydrogen bonds, hydrophobic interactions.134Y is included in local environment of heme 4 in haONRs |
| 251V | V(100%) | T(44%), E(22%) | Core amino acids within subunit hydrogen bonds, hydrophobic interactions in haONRs |
| 222L | L (100%) | T (44%), V(33%) | Core amino acids within subunit hydrophobic interactions and hydrogen bonds |
| 330W | W(100%) | L(44%), F(22%) | Core amino acids within subunit hydrophobic interactions, hydrogen bonds and stacking interactions in haONRs. 330W is included in FFW motif in local environment hemes 6 and 7 in haONRs |
| 200M | M(100%) | L(72%) | Core amino acids within subunit hydrogen bonds, hydrophobic interaction and Aromatic-Sulphur interaction, Y199-M200 in haONRs |
| 369W | W(100%) | A(39%), F(22%) | Core amino acids within subunit hydrophobic interactions |
| 424A | A(100%) | K(33%), M(44%) | Core amino acids within subunit hydrophobic interactions. For GsNiR additional core amino acids within subunit hydrogen bonds |
| 22I | I(100%) | V(56%), I(39%) | Core amino acids within subunit hydrophobic interactions |
| 229F | F(75%), Y(15%) | N(44%), I (28%),  V(17%) | S-aromatic interactions between additional phenylalanine and nearest methionines (F229−M62 for heme 5) in haONRs. 229F is included in the CFMCH motif of heme 5 haONRs |
| 75F | F(100%) | Y(67%) | S-aromatic interactions between additional phenylalanine and nearest methionines (F75−M150 for heme 3) in haONRs. 75F is included in FNSF motif in local environment of hemes 3 and 6 in haONRs |
| 16F | F/Y(50%) | Y(50%), T(28%) | S-aromatic interactions between additional phenylalanine and nearest methionines (F16−M13 for heme 1) in haONRs. 16F is included in CFDCH motif of heme 1 in haONRs |
| 329F | F(50%) | P(100%) | 329F is included in FFW motif in local environment of heme 4 in haONRs |
| 101F | F/Y(50%) | F(44%), Q(33%), | 101F is included in PMF motif in local environment of heme 4 in haONRs |
| 187F | F/N(50%) | Q(56%), S(28%) | 187F is included in CMFCK motif heme 4 in haONRs |
| 155G | G(100%) | K(78%) | Accessible surface. Core amino acids within subunit hydrogen bonds.  For GsNiR additional core amino acids within subunit ionic interactions K144-D147 |
| 204H | H(100%) | D(39%), N(22%),  V(22%) | Accessible surface. Core amino acids within subunit ionic interactions H204-E205, H204-280 in haONRs |
| 52R | R(100%) | K(50%) | Accessible surface. Core amino acids within subunit hydrogen bonds |
| 265S | S(100%) | K(22%), N(39%) | Accessible surface. Core amino acids within subunit hydrogen bonds |
| 284A | A(75%) | K(100%) | Accessible surface. Core amino acids within subunit hydrophobic interactions. For nnONRs additional core amino acids within subunit ionic interaction K260- D233 and Cation-Pi interaction F181- K260 |
| 173F | F(100%) | K(44%), H(33%) | Core amino acids within subunit hydrophobic interactions. For GsNiR additional core amino acids within subunit hydrogen bonds |
| 335D | D(100%) | Q(39%), R(17%) | Accessible surface. Core amino acids within subunit hydrogen bonds, ionic interaction H260-D335 in haONRs |
| 238G | E(75%) | Q(72%), K(22%) | Accessible surface. Core amino acids within subunit hydrogen bonds |
| 266E | E(75%) | G/T(44%) | Accessible surface. Core amino acids within subunit hydrogen bonds |
| 179T | T(75%) | A(50%), M(33%) | Accessible surface. Core amino acids within subunit hydrogen bond |
| 290T | T(25%), E(50%) | K(94%) | Accessible surface. Core amino acids within subunit hydrogen bonds |
| 317R | R(75%) | K(61%), P(33%) | Accessible surface |
| 220R | R(100%) | K(56%), R(33%) | Accessible surface. Core amino acids within subunit hydrogen bonds |
| 148D | D(100%) | G(33%), L(28%) | Accessible surface. Core amino acids within subunit hydrogen bonds and salt bridges |
| 474Y | Y/T(50%) | E(50%), S(44%) | Accessible surface. Core amino acids within subunit hydrogen bonds and hydrophobic interactions |
| 375R | R(50%) | K(61%) | Accessible surface. Core amino acids within subunit hydrogen bonds and salt bridges |
| 164D | D/E(50%) | G(44%), Q(28%) | Accessible surface. Core amino acids within subunit hydrogen bonds and salt bridges |
| 35N | N (100%) | S(28%), A(44%) | Core amino acids within subunit hydrogen bonds |
| 287L | L (100%) | V(44%), I(28%) | Core amino acids within subunit hydrophobic interactions and hydrogen bonds |
| 10V | V(100%) | D(39%)A, (22%) | Hydrophobic interactions in dimer contact in haONRs |
| 38H | H(75%) | S/V(28%), T(33%) | T33(A)-H38(B) hydrogen bond in dimer contact in haONRs |
| 28V | V/R(50%) | G(72%) | N6(A)-V28(B) hydrogen bond in dimer contact in haONRs |
| 27T | V(75%) | E(44%), A(28%) | K8(A)-T27(B) hydrogen bond in dimer contact in haONRs |
| 65E | E/R(50%) | A(67%), Q(17%) | E65K(A) - K394(B) salt bridge in dimer contact in haONRs |
| 330W | W(100%) | L(61%), F(22%) | 330W is included in FFW motif in local environment of hemes 6 and 7 in haONRs |
| 259P | P(100%) | Y(44%), W(44%) | Core amino acids within subunit hydrophobic interaction |
| 92A | A(100%) | G(44%), K(44%) | Core amino acids within subunit hydrophobic interaction |
| 439I | I(100%) | M(56%) | Core amino acids within subunit hydrogen bonds |
| 482W | W(100%) | Y(78%) | Core amino acids within subunit hydrophobic interactions, hydrogen bonds and stacking interactions |
| 419W | W(100%) | K(44%), S(22%) | Core amino acids within subunit hydrogen bonds, T147(C)-W419(A) in trimer contact of haONRs. 419W is involved in hydrophobic interactions with the CLNCH heme-binding motif of heme 8 |
| 440V | V/M/A/N(25%) | R(100%) | P94(A)-440(E), P94(A)-440(C), P94(C)-440(E) hydrophobic interactions in trimer contact in haONRs |
| 506S | T(75%) | A(44%), K(28%) | hydrogen bonds S175(A)/Q176(A)–S506(E)) in trimer contact in haONRs |
| 88R | R,K(50%) | R(100%) | Hydrogen bonds R88(A)–Y430(E) and Cation-Pi interactions R88(A)–Y430(E),Y434(E) in trimer contact in haONRs |
