# Supplementary material for: Structural adaptations of octaheme nitrite reductases from haloalkaliphilic Thioalkalivibrio bacteria to alkaline pH and high salinity
Source: PLoS One. 2017 May 16;12(5):e0177392. doi: 10.1371/journal.pone.0177392 (PMC5433712; doi:10.1371/journal.pone.0177392)
Supplement: S3 Table — (DOC) [file pone.0177392.s003.doc]

S3. Comparison of hydrophobic core in TvNiR and GsNiR

| **The composition of hydrophobic core of hexamer, percentage of number of atoms in the core (for the residues); %** | **GsNiR** | **TvNiR** |
| --- | --- | --- |
| **K** | **10.4** | **4.9** |
| **A** | **3.1** | **4.1** |
| **V** | **6.3** | **8.9** |
| **W** | **6.0** | **7.8** |
| **F** | **11.2** | **14.8** |
| **I** | **6.8** | **4.6** |
| **L** | **10.2** | **10.0** |
| **M** | **4.8** | **5.8** |
| **H** | **9.7** | **10.3** |
| **Y** | **10.0** | **7.7** |
| **D** | **1.8** | **1.4** |
| **E** | **3.6** | **3.4** |
| **R** | **4.0** | **3.4** |
| **Q** | **1.0** | **2.7** |
| **T** | **2.1** | **2.1** |
| **P** | **1.9** | **3.0** |
| **S** | **-** | **-** |
| **G** | **-** | **-** |
| **C** | **3.2** | **2.8** |
| **N** | **1.0** | **2.7** |
| **Total number of atoms in the core; %** | **12.5** | **13.0** |
| **Number of atoms in the entire protein** | **48317.0** | **51644.0** |
| **Surface area of the entire protein, Å2** | **100474.7** | **84396** |
| **Core density, kg / m3** | **2248.6** | **2248.8** |
| **Core volume, сm3/mol** | **33656.3** | **16688.5** |
