# Supplementary material for: Structural adaptations of octaheme nitrite reductases from haloalkaliphilic Thioalkalivibrio bacteria to alkaline pH and high salinity
Source: PLoS One. 2017 May 16;12(5):e0177392. doi: 10.1371/journal.pone.0177392 (PMC5433712; doi:10.1371/journal.pone.0177392)
Supplement: S4 Table — (DOC) [file pone.0177392.s004.doc]

S4. Amino acid substitutions between GsNiR and TvNiR

| Substitutions on the solvent-accessible surfaceGsNiR-TvNiR | | Substitutions in the contacts (dimer, trimer) GsNiR-TvNiR | Substitutions in the core  GsNiR-TvNiR |
| --- | --- | --- | --- |
| G13-D17  V18-I22  K19-E23  A20-D24  L39--  K40-T43  L43-V46  A44-E47  Y46-A49  - -R52  - -R53  T48-E56  K49-R57  N53-R61  N74-R82  К134 -N142  W137-D148  G143-R154  K144 -G155  --D164  --E166  --D169  G153-R171  K154 -R172  К155 -F173  M161-T179  K178 -D196  К180 -A198  F181-Y199  К185 -E203  D186-H204  P187-E205  К188 -A206  D196-E214  I197-V215  A199-E217  K202 -R220  Q220-G238  E233-V251  K234 -D252  D235-R253  T237-L255  F241-D261  A242-V263  N244-S265  G245-E266  K246 -Q267 | K250 -T271  I252-V273  --R279  D256 -E280  G257-D281  K260-A284  E265-D289  K266-T290  A284-P308  K293 - R317  Q299 -R323  K310 -F334  A314-E338  K317 -Q341  K318 -E342  L319-I343  K325 -R349  I328-T352  A352-N376  K365 -Q389  K367 -E391  K373-T397  I378-R402  A381-R405  K386 -G410  K394 -E418  K444 -Q468  K448-L472  E450-Y474  E451-D475  E471-D495  D475 -E499  A486 -E510  K489 -S513  E496-D520 --------V523  K498 -S525 | Dimer  T1-N6  G2-L7  A3-K8  K4-P9  G5-V10  R8-A12  E23-T27  G24-V28  R29-T33  A31-N35  S34-H38  A57-E65  G60-A68  S61-I69  N65-A73  Y67-F75  S68-N76  Y75-H83  V263-L287  K367-E391  D368-N392  P393-A417 | K4-P9  L21-M25  K22-H26  L30-V34  Y46-A49  L54-M62  I121-V129  F125-A133  -------W143  K144-G155  I148-V159  K155-F173  K180-A198  K188-A206  I197-V215  I211-F229  H212-M230  I232-V250  L249-M270  V250-T271  I252-V273  K260-A284  L271-V295  L306-W330  K307-A331  K310-F334  K400-A424  R408- K432  Q424- K448  L425-M449  I437-V461 |
|  |  | Trimer  G84-A92  T103-A111  Y132-F140  K134-N142  G135-Q144  A136-T147  P157-S175  E158-Q176  F241-D261  R332 -A356  R379 -T403  S395-W419  Q403-A427  G406-Y430  T407-I431  R412-H436  M415-I439  R416-V440  T478-M502  A482-S506 |  |
