# Supplementary material for: Structural adaptations of octaheme nitrite reductases from haloalkaliphilic Thioalkalivibrio bacteria to alkaline pH and high salinity
Source: PLoS One. 2017 May 16;12(5):e0177392. doi: 10.1371/journal.pone.0177392 (PMC5433712; doi:10.1371/journal.pone.0177392)
Supplement: S1 Appendix — (DOC) [file pone.0177392.s005.doc]

>pdb|2OT4|A Chain A, Structure Of A Hexameric Multiheme C Nitrite Reductase From The Extremophile Bacterium Thiolkalivibrio Nitratireducens >pdb|2OT4|B Chain B, Structure Of A Hexameric Multiheme C Nitrite Reductase From The Extremophile Bacterium Thiolkalivibrio Nitratireducens

EPGENLKPVDAMQCFDCHTQIEDMHTVGKHATVNCVHCHDATEHVETASSRRMGERPVTRMDLEACATCHTAQFNSFVEV

RHESHPRLEKATPTSRSPMFDKLIAGHGFAFEHAEPRSHAFMLVDHFVVDRAYGGRFQFKNWQKVTDGMGAVRGAWTVLT

DADPESSDQRRFLSQTATAANPVCLNCKTQDHILDWAYMGDEHEAAKWSRTSEVVEFARDLNHPLNCFMCHDPHSAGPRV

VRDGLINAVVDRGLGTYPHDPVKSEQQGMTKVTFQRGREDFRAIGLLDTADSNVMCAQCHVEYNCNPGYQLSDGSRVGMD

DRRANHFFWANVFDYKEAAQEIDFFDFRHATTGAALPKLQHPEAETFWGSVHERNGVACADCHMPKVQLENGKVYTSHSQ

RTPRDMMGQACLNCHAEWTEDQALYAIDYIKNYTHGKIVKSEYWLAKMIDLFPVAKRAGVSEDVLNEARELHYDAHLYWE

WWTAENSVGFHNPDQARESLMTSISKSKEAVSLLNDAIDAQVASR

>pdb|3F29|A Chain A, Structure Of The Thioalkalivibrio Nitratireducens Cytochrome C Nitrite Reductase In Complex With Sulfite >pdb|3F29|B Chain B, Structure Of The Thioalkalivibrio Nitratireducens Cytochrome C Nitrite Reductase In Complex With Sulfite >pdb|2ZO5|A Chain A, Structure Of The Thioalkalivibrio Nitratireducens Cytochrome C Nitrite Reductase In A Complex With Azide >pdb|2ZO5|B Chain B, Structure Of The Thioalkalivibrio Nitratireducens Cytochrome C Nitrite Reductase In A Complex With Azide >pdb|3D1I|A Chain A, Structure Of The Thioalkalivibrio Nitratireducens Cytochrome C Nitrite Reductase In A Complex With Nitrite >pdb|3D1I|B Chain B, Structure Of The Thioalkalivibrio Nitratireducens Cytochrome C Nitrite Reductase In A Complex With Nitrite >pdb|3GM6|A Chain A, Structure Of The Thioalkalivibrio Nitratireducens Cytochrome C Nitrite Reductase In Complex With Phosphate >pdb|3GM6|B Chain B, Structure Of The Thioalkalivibrio Nitratireducens Cytochrome C Nitrite Reductase In Complex With Phosphate >pdb|3FO3|A Chain A, Structure Of The Thioalkalivibrio Nitratireducens Cytochrome C Nitrite Reductase Reduced By Sodium Dithionite (Sulfite Complex) >pdb|3FO3|B Chain B, Structure Of The Thioalkalivibrio Nitratireducens Cytochrome C Nitrite Reductase Reduced By Sodium Dithionite (Sulfite Complex) >pdb|3MMO|A Chain A, Structure Of The Thioalkalivibrio Nitratireducens Cytochrome C Nitrite Reductase In Complex With Cyanide >pdb|3MMO|B Chain B, Structure Of The Thioalkalivibrio Nitratireducens Cytochrome C Nitrite Reductase In Complex With Cyanide >pdb|3LG1|A Chain A, Structure Of The Thioalkalivibrio Nitratireducens Cytochrome C Nitrite Reductase Reduced By Sodium Borohydride (In Complex With Sulfite) >pdb|3LG1|B Chain B, Structure Of The Thioalkalivibrio Nitratireducens Cytochrome C Nitrite Reductase Reduced By Sodium Borohydride (In Complex With Sulfite) >pdb|3LGQ|A Chain A, Structure Of The Thioalkalivibrio Nitratireducens Cytochrome C Nitrite Reductase In Complex With Sulfite (Modified Tyr-303) >pdb|3LGQ|B Chain B, Structure Of The Thioalkalivibrio Nitratireducens Cytochrome C Nitrite Reductase In Complex With Sulfite (Modified Tyr-303) >pdb|4Q17|A Chain A, Free Form Of Tvnir, Middle Dose Data Set >pdb|4Q17|B Chain B, Free Form Of Tvnir, Middle Dose Data Set >pdb|4Q1O|A Chain A, Free Form Of Tvnir, High Dose Data Set >pdb|4Q1O|B Chain B, Free Form Of Tvnir, High Dose Data Set

EPGENLKPVDAMQCFDCHTQIEDMHTVGKHATVNCVHCHDATEHVETASSRRMGERPVTRMDLEACATCHTAQFNSFVEV

RHESHPRLEKATPTSRSPMFDKLIAGHGFAFEHAEPRSHAFMLVDHFVVDRAYGGRFQFKNWQKVTDGMGAVRGAWTVLT

DADPESSDQRRFLSQTATAANPVCLNCKTQDHILDWAYMGDEHEAAKWSRTSEVVEFARDLNHPLNCFMCHDPHSAGPRV

VRDGLINAVVDRGLGTYPHDPVKSEQQGMTKVTFQRGREDFRAIGLLDTADSNVMCAQCHVEYNCNPGYQLSDGSRVGMD

DRRANHFFWANVFDYKEAAQEIDFFDFRHATTGAALPKLQHPEAETFWGSVHERNGVACADCHMPKVQLENGKVYTSHSQ

RTPRDMMGQACLNCHAEWTEDQALYAIDYIKNYTHGKIVKSEYWLAKMIDLFPVAKRAGVSEDVLNQARELHYDAHLYWE

WWTAENSVGFHNPDQARESLMTSISKSKEAVSLLNDAIDAQVASR

>WP_015257125.1 eight-heme nitrite reductase precursor [Thioalkalivibrio nitratireducens] >L0DSL2.1 RecName: Full=Cytochrome c-552; AltName: Full=Cytochrome c nitrite reductase; AltName: Full=TvNiR; Flags: Precursor >CAI56199.2 eight-heme nitrite reductase precursor [Thioalkalivibrio nitratireducens DSM 14787] >AGA31970.1 eight-heme nitrite reductase precursor [Thioalkalivibrio nitratireducens DSM 14787]

MNDLNRLGRVGRWIAGAACLFLASAAHAEPGENLKPVDAMQCFDCHTQIEDMHTVGKHATVNCVHCHDATEHVETASSRR

MGERPVTRMDLEACATCHTAQFNSFVEVRHESHPRLEKATPTSRSPMFDKLIAGHGFAFEHAEPRSHAFMLVDHFVVDRA

YGGRFQFKNWQKVTDGMGAVRGAWTVLTDADPESSDQRRFLSQTATAANPVCLNCKTQDHILDWAYMGDEHEAAKWSRTS

EVVEFARDLNHPLNCFMCHDPHSAGPRVVRDGLINAVVDRGLGTYPHDPVKSEQQGMTKVTFQRGREDFRAIGLLDTADS

NVMCAQCHVEYNCNPGYQLSDGSRVGMDDRRANHFFWANVFDYKEAAQEIDFFDFRHATTGAALPKLQHPEAETFWGSVH

ERNGVACADCHMPKVQLENGKVYTSHSQRTPRDMMGQACLNCHAEWTEDQALYAIDYIKNYTHGKIVKSEYWLAKMIDLF

PVAKRAGVSEDVLNQARELHYDAHLYWEWWTAENSVGFHNPDQARESLMTSISKSKEAVSLLNDAIDAQVASR

>pdb|4Q0T|A Chain A, Free Form Of Tvnir, Low Dose Data Set >pdb|4Q0T|B Chain B, Free Form Of Tvnir, Low Dose Data Set

PGENLKPVDAMQCFDCHTQIEDMHTVGKHATVNCVHCHDATEHVETASSRRMGERPVTRMDLEACATCHTAQFNSFVEVR

HESHPRLEKATPTSRSPMFDKLIAGHGFAFEHAEPRSHAFMLVDHFVVDRAYGGRFQFKNWQKVTDGMGAVRGAWTVLTD

ADPESSDQRRFLSQTATAANPVCLNCKTQDHILDWAYMGDEHEAAKWSRTSEVVEFARDLNHPLNCFMCHDPHSAGPRVV

RDGLINAVVDRGLGTYPHDPVKSEQQGMTKVTFQRGREDFRAIGLLDTADSNVMCAQCHVEYNCNPGYQLSDGSRVGMDD

RRANHFFWANVFDYKEAAQEIDFFDFRHATTGAALPKLQHPEAETFWGSVHERNGVACADCHMPKVQLENGKVYTSHSQR

TPRDMMGQACLNCHAEWTEDQALYAIDYIKNYTHGKIVKSEYWLAKMIDLFPVAKRAGVSEDVLNQARELHYDAHLYWEW

WTAENSVGFHNPDQARESLMTSISKSKEAVSLLNDAIDAQVASR

>pdb|4Q4U|A Chain A, Tvnir In Complex With Sulfite, Low Dose Data Set >pdb|4Q4U|B Chain B, Tvnir In Complex With Sulfite, Low Dose Data Set >pdb|4Q5C|A Chain A, Tvnir In Complex With Sulfite, Middle Dose Data Set >pdb|4Q5C|B Chain B, Tvnir In Complex With Sulfite, Middle Dose Data Set >pdb|4Q5B|A Chain A, Tvnir In Complex With Sulfite, High Dose Data Set >pdb|4Q5B|B Chain B, Tvnir In Complex With Sulfite, High Dose Data Set

NLKPVDAMQCFDCHTQIEDMHTVGKHATVNCVHCHDATEHVETASSRRMGERPVTRMDLEACATCHTAQFNSFVEVRHES

HPRLEKATPTSRSPMFDKLIAGHGFAFEHAEPRSHAFMLVDHFVVDRAYGGRFQFKNWQKVTDGMGAVRGAWTVLTDADP

ESSDQRRFLSQTATAANPVCLNCKTQDHILDWAYMGDEHEAAKWSRTSEVVEFARDLNHPLNCFMCHDPHSAGPRVVRDG

LINAVVDRGLGTYPHDPVKSEQQGMTKVTFQRGREDFRAIGLLDTADSNVMCAQCHVEYNCNPGYQLSDGSRVGMDDRRA

NHFFWANVFDYKEAAQEIDFFDFRHATTGAALPKLQHPEAETFWGSVHERNGVACADCHMPKVQLENGKVYTSHSQRTPR

DMMGQACLNCHAEWTEDQALYAIDYIKNYTHGKIVKSEYWLAKMIDLFPVAKRAGVSEDVLNQARELHYDAHLYWEWWTA

ENSVGFHNPDQARESLMTSISKSKEAVSLLNDAIDAQVASR

>pdb|3UU9|A Chain A, Structure Of The Free Tvnirb Form Of Thioalkalivibrio Nitratireducens Cytochrome C Nitrite Reductase >pdb|3UU9|B Chain B, Structure Of The Free Tvnirb Form Of Thioalkalivibrio Nitratireducens Cytochrome C Nitrite Reductase

NLKPVDAMQCFDCHTQIEDMHTVGKHATVNCVHCHDATEHVETASSRRMGERPVTRMDLEACATCHTAQFNSFVEVRHES

HPRLEKATPTSRSPMFDKLIAGHGFAFEHAEPRSHAFMLVDHFVVDRAYGGRFQFKNWQKVTDGMGAVRGAWTVLTDADP

ESSDQRRFLSQTATAANPVCLNCKTQDHILDWAYMGDEHEAAKWSRTSEVVEFARDLNHPLNCFMCHDPHSAGPRVVRDG

LINAVVDRGLGTYPHDPVKSEQQGMTKVTFQRGREDFRAIGLLDTADSNVMCAQCHVEYNCNPGYQLSDGSRVGMDDRRA

NHFFWANVFDYKEAAQEIDFFDFRHATTGAALPKLQHPEAETFWGSVHERNGVACADCHMPKVQLENGKVYTSHSQRTPR

DMMGQACLNCHAEWTEDQALYAIDYIKNYTHGKIVKSEYWLAKMIDLFPVAKRAGVSEDVLNQARELHYDAHLYWEWWTA

ENSVGFHNPDQARESLMTSISKSKEAVSLLNDAIDAQVAS

>pdb|3RKH|A Chain A, Structure Of The Thioalkalivibrio Nitratireducens Cytochrome C Nitrite Reductase In A Complex With Nitrite (Full Occupancy) >pdb|3RKH|B Chain B, Structure Of The Thioalkalivibrio Nitratireducens Cytochrome C Nitrite Reductase In A Complex With Nitrite (Full Occupancy) >pdb|4L3X|A Chain A, Nitrite Complex Of Tvnir, First Middle Dose Data Set >pdb|4L3X|B Chain B, Nitrite Complex Of Tvnir, First Middle Dose Data Set >pdb|4L3Y|A Chain A, Nitrite Complex Of Tvnir, High Dose Data Set (no Complex) >pdb|4L3Y|B Chain B, Nitrite Complex Of Tvnir, High Dose Data Set (no Complex) >pdb|4L3Z|A Chain A, Nitrite Complex Of Tvnir, Second Middle Dose Data Set >pdb|4L3Z|B Chain B, Nitrite Complex Of Tvnir, Second Middle Dose Data Set >pdb|4L38|A Chain A, Nitrite Complex Of Tvnir, Low Dose Data Set >pdb|4L38|B Chain B, Nitrite Complex Of Tvnir, Low Dose Data Set

NLKPVDAMQCFDCHTQIEDMHTVGKHATVNCVHCHDATEHVETASSRRMGERPVTRMDLEACATCHTAQFNSFVEVRHES

HPRLEKATPTSRSPMFDKLIAGHGFAFEHAEPRSHAFMLVDHFVVDRAYGGRFQFKNWQKVTDGMGAVRGAWTVLTDADP

ESSDQRRFLSQTATAANPVCLNCKTQDHILDWAYMGDEHEAAKWSRTSEVVEFARDLNHPLNCFMCHDPHSAGPRVVRDG

LINAVVDRGLGTYPHDPVKSEQQGMTKVTFQRGREDFRAIGLLDTADSNVMCAQCHVEYNCNPGYQLSDGSRVGMDDRRA

NHFFWANVFDYKEAAQEIDFFDFRHATTGAALPKLQHPEAETFWGSVHERNGVACADCHMPKVQLENGKVYTSHSQRTPR

DMMGQACLNCHAEWTEDQALYAIDYIKNYTHGKIVKSEYWLAKMIDLFPVAKRAGVSEDVLNQARELHYDAHLYWEWWTA

ENSVGFHNPDQARESLMTSISKSKEAVSLLNDAIDAQVAV

>pdb|3SCE|A Chain A, Structure Of The Thioalkalivibrio Nitratireducens Cytochrome C Nitrite Reductase With A Covalent Bond Between The Ce1 Atom Of Tyr303 And The Cg Atom Of Gln360 (Tvnirb) >pdb|3SCE|B Chain B, Structure Of The Thioalkalivibrio Nitratireducens Cytochrome C Nitrite Reductase With A Covalent Bond Between The Ce1 Atom Of Tyr303 And The Cg Atom Of Gln360 (Tvnirb) >pdb|3S7W|A Chain A, Structure Of The Tvnirb Form Of Thioalkalivibrio Nitratireducens Cytochrome C Nitrite Reductase With An Oxidized Gln360 In A Complex With Hydroxylamine >pdb|3S7W|B Chain B, Structure Of The Tvnirb Form Of Thioalkalivibrio Nitratireducens Cytochrome C Nitrite Reductase With An Oxidized Gln360 In A Complex With Hydroxylamine >pdb|3OWM|A Chain A, Structure Of The Thioalkalivibrio Nitratireducens Cytochrome C Nitrite Reductase In A Complex With Hydroxylamine >pdb|3OWM|B Chain B, Structure Of The Thioalkalivibrio Nitratireducens Cytochrome C Nitrite Reductase In A Complex With Hydroxylamine

NLKPVDAMQCFDCHTQIEDMHTVGKHATVNCVHCHDATEHVETASSRRMGERPVTRMDLEACATCHTAQFNSFVEVRHES

HPRLEKATPTSRSPMFDKLIAGHGFAFEHAEPRSHAFMLVDHFVVDRAYGGRFQFKNWQKVTDGMGAVRGAWTVLTDADP

ESSDQRRFLSQTATAANPVCLNCKTQDHILDWAYMGDEHEAAKWSRTSEVVEFARDLNHPLNCFMCHDPHSAGPRVVRDG

LINAVVDRGLGTYPHDPVKSEQQGMTKVTFQRGREDFRAIGLLDTADSNVMCAQCHVEYNCNPGYQLSDGSRVGMDDRRA

NHFFWANVFDYKEAAQEIDFFDFRHATTGAALPKLQHPEAETFWGSVHERNGVACADCHMPKVQLENGKVYTSHSQRTPR

DMMGQACLNCHAEWTEDQALYAIDYIKNYTHGKIVKSEYWLAKMIDLFPVAKRAGVSEDVLNQARELHYDAHLYWEWWTA

ENSVGFHNPDQARESLMTSISKSKEAVSLLNDAIDAQVA

>pdb|3TTB|A Chain A, Structure Of The Thioalkalivibrio Paradoxus Cytochrome C Nitrite Reductase In Complex With Sulfite >pdb|3TTB|B Chain B, Structure Of The Thioalkalivibrio Paradoxus Cytochrome C Nitrite Reductase In Complex With Sulfite >pdb|3SXQ|A Chain A, Structure Of A Hexameric Multiheme C Nitrite Reductase From The Extremophile Bacterium Thiolkalivibrio Paradoxus >pdb|3SXQ|B Chain B, Structure Of A Hexameric Multiheme C Nitrite Reductase From The Extremophile Bacterium Thiolkalivibrio Paradoxus

GSGDQLKPVDALQCYDCHTQIEDMHVVGKHATVNCVHCHDATEHVETASARRMGERPVTHTSPEACASCHTAQFNSFASV

RHESHPREEKANPRSRSPKFDTLIGAHGFSLEHAEPRSHAFMLVDHFIVDRAYGGRFQYKSWQNVTDGLGAVRGAWTVIE

DMDPTTSDQRRFLAQTATAANPVCLNCKTQDHILDWAYMGDEHDAAKWARTSKVVDFARDLHHPVNCYMCHDPHSTEPRV

VRDALIHAVVDQGLGTYPYDEAKSEHVTLTPVTFQRGGEDFRKIGLLNVADSNLMCGQCHVEYNCNPGFQQSDGAPVGMD

DRRTNHFFWANVFDYAEAAKEIDFFDFTHVTTGAPLPKLQHPELETFWGSTHERNGVTCADCHMPRVKLENGKEYTMHSP

RTPRDMMNRACLNCHDGWTEAEAEYAIDYIKNYTHGKIMKAEFWLARMIDLFPVAKRAGVSEDVLNEVRALHYDAHLHWE

WWTAENSVGFHNPDQARESLMKSITKSKEGVGKLDAAIDAAVAAN

>WP_006746849.1 cytochrome c [Thioalkalivibrio paradoxus] >ADV18468.1 eight-heme nitrite reductase [Thioalkalivibrio paradoxus] >AHE99576.1 cytochrome C [Thioalkalivibrio paradoxus ARh 1]

MNDLNRLGRVGRWVAGAACLFLASAAHAGSGDQLKPVDALQCYDCHTQIEDMHVVGKHATVNCVHCHDATEHVETASARR

MGERPVTHTSPEACASCHTAQFNSFASVRHESHPREEKANPRSRSPKFDTLIGAHGFSLEHAEPRSHAFMLVDHFIVDRA

YGGRFQYKSWQNVTDGLGAVRGAWTVIEDMDPTTSDQRRFLAQTATAANPVCLNCKTQDHILDWAYMGDEHDAAKWARTS

KVVDFARDLHHPVNCYMCHDPHSTEPRVVRDALIHAVVDQGLGTYPYDEAKSEHVTLTPVTFQRGGEDFRKIGLLNVADS

NLMCGQCHVEYNCNPGFQQSDGAPVGMDDRRTNHFFWANVFDYAEAAKEIDFFDFTHVTTGAPLPKLQHPELETFWGSTH

ERNGVTCADCHMPRVKLENGKEYTMHSPRTPRDMMNRACLNCHDGWTEAEAEYAIDYIKNYTHGKIMKAEFWLARMIDLF

PVAKRAGVSEDVLNEVRALHYDAHLHWEWWTAENSVGFHNPDQARESLMKSITKSKEGVGKLDAAIDAAVAAN

>OGW23475.1 cytochrome C [Nitrospirae bacterium GWB2_47_37]

MSKKIYVLFTVVFVLALVGGIYFSTADARKDMPAGKPVKAEECYSCHSDIKDFHAKGKHAKVNCVECHEGLDKHMKEGAN

AKPMTKTDHATCGKCHKEQYESFVAVNLESKAKVEKATFKSRSPLFDKLMMPHGFTKEHAEPRSHIFMLTDHLTVDRGYG

GRFQLKDWKKITDAKGAEKSAWEVLVDKDPSSSAQKAFIPQSATAANPVCLTCKTQDHIMKWKYMGDKDPRAQWDRTSKV

VDFARDLQHPINCYACHDPHSAQPRVVRDALIEAVVDRGEGTYPYDKEKSKKITMKKVTFRDFRAIGILNKADSNLMCAQ

CHVEYNCNPGHDPKTGASIGMGDRRTNYFPWVNVFDINKRYDEIGFKDFKHGITGASLTKMQHPEAESFWGSKHEKAGVE

CKDCHMPRVKKGAKTYTWHGQKSARYMEKATCVKCHPSWTEKEADYQIDAIQNYIKGKLTKAEFWLGQFIDTFAKAKKAG

VSEDVLKEARKLHDTAHSYWEWWTAENSDGFHNPDGARESLARSIDASQKGIKVLNDAMAAKTAAK

>SDI64766.1 Formate-dependent nitrite reductase, cytochrome c552 subunit [Propionivibrio dicarboxylicus]

MRTKKIKAGLVGVLAICATVLGIGVAEARQPDPAVVKQQDQVTQKTLEQVRGALPKTAMAKDKQADVAACYSCHKDIKEF

HVSSKHASVNCATCHTNFDEHVAKDGKAPIATRTDHAVCGTCHQPQYESFLSVNYESKARIEKATYKGRSPLFDKLMAPH

GFTKEHTEPRSHIFMLLDQLLIDRSYGGRFQLNDWSMLADGKGAETQLWSALLKDADPSSSDQKVFMPQTATASNPVCLN

CKTQDHILKWKYMGDPDPKAKWSRTSKVVDFVRDLKHPMNCYTCHDPHSTAPRVVRDALIEAVVDRGMGTYPQDKAKSGK

VTMTKVTFRDGFRAIGVLNKPDSNLMCAQCHVEYNCNPGFDTKTGELSVTMADRRANHYFWSNVFDYKAAAEKISFKDFK

HATTGALLSKIQHPEAETFWGSAHERNNVECKSCHMPKIKQDGKTYTSHFQRSPRYNVKDTCLKCHNDMNEQQAVYTIDS

IQNYTRGKLAKAEYWLAQLIDTFPKARAAGVPDEAIKKAQAHHDQAHIYWEWWTAENSDGFHNPGAARESLTRSMDASQA

GIKVLNDAIAALAKK

>OGW30387.1 cytochrome C [Nitrospirae bacterium GWF2_44_13] >OGW36032.1 cytochrome C [Nitrospirae bacterium GWD2_44_7] >OGW66430.1 cytochrome C [Nitrospirae bacterium RIFOXYA2_FULL_44_9]

MKRRFFIAFMAALAISLLIGAYYTSADAEKAKPYAGTTAKKAMPAEKGVKAETCYSCHSDIKDFHAKGKHAKVNCSNCHE

GLNSHVKDAMTKPVTRTDHAVCGKCHKEQYESFVSVNLESKAKVEKATFKSRSPLFDKLMMPHGFVKEHAEPRSHIFMLV

DHLTIDRGYGGRFQLKDWTKILDAKGAEKSAWGILMDKDPSTSDQKAFIPQSATAANSVCLQCKSQDNILRWKYMGDKDP

AAKWDRTSKVVDLAKDLKHPMNCYMCHDPHSTQPRVVRDGLIEAVVDRGKGTYPYDKEKSSKITMKKVMFRDFRAIGILS

KADSNLMCAQCHVEYNCNPGHDPKTGKAIGMTDRRTNYFPWANVFDINKTYEELGFKDFKHAVTGASLTKMQHPETETFW

GSKHEKAGVECKDCHMPRVKKAGKTVTFHGQRSARYMLKDTCLNCHKGWTVKEAEYQVDAIQNYIKGKLTKAEFWLGQFI

DTFAKAKAKGVSEDVLKDAKKLHDTAHTYWEWWTAENSDGFHNPDAARESLTRSIDASQKGIKILNDAMAPKPAAK

>WP_007293236.1 cytochrome c [delta proteobacterium MLMS-1] >EAT04113.1 cytochrome c family protein [delta proteobacterium MLMS-1]

MSKCKSLLTAVAAFAVAGGISLFGATSVEAKPDFQPVDIDTCYNCHDTTGPGSDIKAFHVRGSHAGINCGYCHTETDPHV

RNFNTKPVTSVDQRTCGQCHAEQFNSMLQTNWDRPAKEEKATPTGRSPMYDKLMRPHGFTREHAEPRSHVFMLIDHLVVD

RGYGGRFDFKSWELIGDAAAAQRGAWEVLYDKEPETNDHKAFREQTANAANPVCMFCKSQDNILSWAYMGDPHPKAEWDR

TSNVVDFARSLNHGLNCFTCHDPHSTEPRITRDAQIQAIVDRGEGTYPYDEAKSERITMEKVEFRDGFRAIGLLSEPDSN

MMCAQCHVTYNCNPGTDAKTGERVTMADQRTNLMPWVNVWDIEEAMFDKFAFYDFRDAVTGAKKIKFQHPEMEVFWGSTH

ERAGVQCQDCHMPRMVSEAGVEYTSHFQTSPRYNLESTCLQCHSDWSAEQAMYTINSVKNYVRGRIAKTEFWLSEFIDTF

VRAQDLGVPESVLEEARGYHTSANTHWEWWVAENSDGFHNPQQARESLARSMTLSQEGIALLNKAIEERR

>BAU23742.1 cytochrome C [Caldimicrobium thiodismutans]

MGMKGKKLKWSMGLPLALLGGALVVGGLYSQVEAKKGVTSPVEKADVQTCYGCHSEIKDLHATSKHKNLNCNVCHSNFGK

HLENPMENKPITRLEHAVCGQCHKDQYETFVSVNLASPAKIEKATSTSRSPLFDNLMRPHGFTREHAEPRSHIFALVDHL

LVDRAYGGRFQLKDWTMLFNAKAAEKSAWEVLKDADPASSDQKIFPPYTPRTAATAANPVCLQCKTTDFILKWAYMGDKH

PKAKWDRTSKVVEMARDAHRTFACVHCHDPHAAKHRVVRDALIEAIVDRGKGTYPYDPEKSKKVTVQKIVFRDFRAIGIL

NKADANIMCAQCHVEYNCNPGFDPKTGQAIPMADRRTNFFPWVNVMDIEKVYDEIGFRDFKHEITGAPLIKLQHPEVETF

WGSKHERAGVTCADCHMPKVKNKQGKIYTFHGQRSVKYVPGRTAVCVNCHKYWTPEQAEYVISGIQNYIRGKMRKAEFWI

SKLVDTYAQAQAVGVSEEVLAQARQKHAQAHHYWEWWTAENSDGFHNPELATASLNKAIQLANDGVLLLEKAIKEKRTSG

K

>WP_068516723.1 cytochrome C [Caldimicrobium thiodismutans]

MGLPLALLGGALVVGGLYSQVEAKKGVTSPVEKADVQTCYGCHSEIKDLHATSKHKNLNCNVCHSNFGKHLENPMENKPI

TRLEHAVCGQCHKDQYETFVSVNLASPAKIEKATSTSRSPLFDNLMRPHGFTREHAEPRSHIFALVDHLLVDRAYGGRFQ

LKDWTMLFNAKAAEKSAWEVLKDADPASSDQKIFPPYTPRTAATAANPVCLQCKTTDFILKWAYMGDKHPKAKWDRTSKV

VEMARDAHRTFACVHCHDPHAAKHRVVRDALIEAIVDRGKGTYPYDPEKSKKVTVQKIVFRDFRAIGILNKADANIMCAQ

CHVEYNCNPGFDPKTGQAIPMADRRTNFFPWVNVMDIEKVYDEIGFRDFKHEITGAPLIKLQHPEVETFWGSKHERAGVT

CADCHMPKVKNKQGKIYTFHGQRSVKYVPGRTAVCVNCHKYWTPEQAEYVISGIQNYIRGKMRKAEFWISKLVDTYAQAQ

AVGVSEEVLAQARQKHAQAHHYWEWWTAENSDGFHNPELATASLNKAIQLANDGVLLLEKAIKEKRTSGK

>WP_013162309.1 cytochrome c [Desulfurivibrio alkaliphilus] >ADH84778.1 formate-dependent nitrite reductase periplasmic cytochrome c552 subunit-like protein [Desulfurivibrio alkaliphilus AHT 2]

MIKNPKSLLGAAVAFALVGGLSLFGASGAQAAAKPVDVNTCYNCHDNTGPGSDIKAFHVRGSHASINCGHCHTETEAHVR

NFNVKPVTSVDQRTCGQCHAEQFNSMLQVNWDRPPKEEKATPTGRTSRYDTLMRPHGFTKEHAEPRSHVFMLVDHLLVDR

AYGGRFQLKSWELIGDGAAAQAGAWNILYDVEPETNNQKAFRGQVATAANPVCMFCKSQDNILNWAYMGDPHPKAKWDRT

SNVVDFARSLNHALNCFTCHDPHSTEPRITRDAQIQAIVDRGEGTYPYDKEKSKRITVEKVEFRDGFRAIGLLSEPDSNM

MCAQCHVEYNCNPGIDAATGERVTMADQRTNLMQWSNVFDFNRRMYEDFKFIDFRNAVTGADQMKIQHPEMEVFWGSKME

QAGVECKDCHMPKMVSKAGVKYTSHFQTSPKYHGYENTCLVCHDDWTPERADYTIHAIKNYVRGKINKAEFWLAELIDTF

VRAKDLGVPESVLAEVRKHHTEANTHWEWWVAENSDGFHNPEQARESLAYSMTQSQQGIALLEKAIEERRKAIAKLR

>KPK87831.1 hypothetical protein AMJ94_16365 [Deltaproteobacteria bacterium SM23_61]

MLWKLWICLFFGWIALSLTVSPAQAQKEKSPSLKPEAAPAEKIDEEICFGCHSEIESLKTKGKHAKAVPCGDCHSNLKAH

LEDTGKNPVTRLGPENCGACHQEQYQTYMTANLKSRAKLEKSTTTSRSPTFDKLMAPHPFTREHAEPRSHAFMLIDYLIV

DRAYGGRFQLKDWTYIDKKGKLWDVVADKGKELPQTAKAGNTVCMTCKTSDHVLKWSYLGDPNPATHLKRGLNPEAVEMA

RNHVQRPMGCIHCHDPHAGKPRVIRDALIEAVVDRGGGTYPYDPKKSKEITVEKIIFKRDGKDFRAIGILSKPDSNLMCA

QCHVEYNCNPGFNPQTGAAIGMDNRLTNYFPWVNAFDLQKRYERVGFKDFRHAVTGAALTKIQHPEVETNWGSKHERAGV

ECKDCHMPRVKLGKGKEYTFHGQRSARYMLKDTCVRCHPEWTAEEAEYQVDAIQNYVRGKMRKAEFWLGELINTFIRAKD

LGVGEEILKKARNEHDRAHILWEWWTAENSDGFHNPQAARQSLTESIIASQKGVEILNKAIGQKTAAK

>OGP78137.1 hypothetical protein A2V86_03055 [Deltaproteobacteria bacterium RBG_16_49_23]

MLKRFFFISFVCCLSLVFYLSYARSAKETVSKEEAACYQCHEEIKSLKAGNKHAPLSCAKCHSKLAEHLKDPEKLPETNL

ELSLCGQCHPSQYETYISVNLKSKAKVEKATTASRSPTFDKLMTPHGFTKEHDEPRSHGFMLTDHFLVDRAYGGRFQLKS

WRDITKVGKLWDVVYDTGKELPQTAKAANTVCLTCKTTDTILKWSYMGDPHPATDLKRGGNPAAIDMAKKYLKNPMGCIQ

CHDPHATKPRVVRDALIQAVVDRGEGTYPYNKDKSREITIKKVEFQRDGKPFRAIGILSRMDSNLLCAQCHVEYNCNPGF

DPKTGAAIGMEDRRTNYFPWVNVLDLQKKYGDIGFKDFRHAVTGAPLTKLQHPETETFWGSKHEKAGIECKDCHMPRVKD

KKGKTFTFHGQRSARYMFRDTCVRCHTYWSQEEAEYQVDAIQNYIRGKMRKAEYWLGQLIETYTRAKDLGVSEDILKGAW

PYHDKAHILWEWWTAENSDGFHNPQMAREALAQSINASQEGIRLLEKAIEEKKK

>OGP29373.1 hypothetical protein A2X91_04860 [Deltaproteobacteria bacterium GWB2_65_81] >OGP80007.1 hypothetical protein A2Z26_08230 [Deltaproteobacteria bacterium RBG_16_66_15]

MTVRAVPKAARPAESPVREEKCFECHDDIQALKKGGKHGKVNCTNCHDGTADHLKDSDRKPTTRVDLETCGGCHPDQYAS

FGTLNLRKPARTEKSLLTERAPNPFWDKLMMGHGFTKEHANPRSHAFMLVDHLIVDRAYGGRFQAKNGWGYVSMPGPVKA

WDVLVDRYPDSKEHKAFLPESAPAANPTCLQCKTQDQILKWKYMGDKDEKAKWDRASNVVEFVKELNNSLNCFMCHDPHA

ARPRIVRDGLIQALTRPEKDTLWHKDPNATKIDVKEFRGGFRKIALLEKYDAKLQCGQCHVEYNCNPGYDPKTGEYSIKA

ADRRTNHFPFKNVLQIYDHYNTLGFRDFKHTLTGGLLWKAQHPEAETFWNSVHDKAGASCDACHMPKVRNAKGKVYTSHW

QTSPRNYLKQTCLTSKCHPKSTEAQAAYEIDSIRNFTKGKMRKAEFWLSALIDKIVEGKKAGLPPEVIKEAQELHQKAHV

LWEWWTAENSDGFHNPALARESLTRSVEESRKGITLVNDALERKTASK

>OGP78422.1 hypothetical protein A2Z13_07850 [Deltaproteobacteria bacterium RBG_16_64_85]

MKRSALVWIAMLVGAATYVLSSTAMAVKESPVKEEKCFDCHDEIQALKTGGKHAKINCVSCHSGTAGHLADSDKKPVTRV

DLEACGGCHKDQYESFGTLNLKKPARVEKSLLTERSPNPFWDKLMMGHGFTKEHAAPRSHTFMLVDHLIVDRAYGGRFQS

KNGWGYVSTTGVLKAWDLLEDRYPESKEHKPFIPESAAAANPTCLQCKTQDQILKWKFLGDKDSKAKWDRTSNVVEFVKD

LSNAMNCFMCHDPHAAKPRIVRDGLIGALTRPEKDTLWHKDPRATKIDVREFRGGFRKIALLEKYDAKLQCGQCHVEYNC

NPGYDPKTGEYSIKAADPRTNHFPFKDVFQIYDHYNALGFRDFKHPLTGGLLWKAQHPEAETYWNSRHDKAGANCNDCHM

PKVKNKKGKVFTSHWQTSPRNYVRETCLTAKCHPKLTEAQAAYEIDSVKSFIKGKMRKAEFWLSALIDKIVEGKKAGLDA

DILKEAQEQHQKAHVLWEWWTAENSDGFHNPDMARQSLTKSVEESRKGIKIIAEALEKKVAAK

>WP_011736832.1 cytochrome c [Pelobacter propionicus] >ABL00597.1 cytochrome c family protein [Pelobacter propionicus DSM 2379]

MRKNTLACAAAVIGTLALLSLPVLTTAAKAKPANDGRAQCYECHEEVKALKEGSRHASLSCATCHGKMDEHMNDPESSRP

VTVIDQALCARCHKQQYGSFVTVNYEAHARREKGIPTGRSPMQDKLLAGHGFTFEHAEPRGHAFMVTDQFIVDRFQGGRF

QYRDGWKGVDRTGKTWDVLTDTGRKLPETAMAGNPTCIQCKSSDHILRWKFLGDRDPKAKWDRGSDVVAIAKDTNNPMGC

IHCHDPHGTQPRVVRDALIQAIEKEPATNIFARNGRTDLRMISFRDGFRKIGVMEKVDSRMMCAQCHVEYNCNAGNQWSD

GAKVGFDDRRTNHFPLRNSLQLLKHYRELDFYDFKHAITGARLVKFQHPEAETYAGSVHDRAGVQCHQCHMPQQKGKNGK

SFSNHGVIRPKNHVRESCLGCHPDSTVERKLYQIEAVRNYISGKMRKSEYWLGQLIDSYVAAQRMGVTESVLAQAREKHE

EAHVLWEYWTAENSDGFHNPDLARESLAGSIAASKAGVRILNDAMQVARKGDGK

>WP_045667907.1 cytochrome c [Geobacter sulfurreducens] >AJY70082.1 cytochrome C [Geobacter sulfurreducens]

MWKKRLAVLAVATGAAVALSIPALSTAAKGKPAATGAKGDGRETCYGCHEEVKALKEGSKHARLACDSCHDKLKEHLANY

ETKPGTNLDPAKCGSCHKNEYSSFFTVNYEAQPRKEKGIPTGRSPMQDKLLAGHGFTFEHNEPRGHAFMVVDQFIVDRFQ

GGRFQYKKGAWGMDATGKAWDILTDTGKKLPETAMAGNPTCIQCKTSDHILKWKFMGDKDPKATWDRSSDIVAVAKDTQN

PVGCIHCHDPHGTHPRVVRDGLIQAIENDPTANIFAKNGKTDLKVISFRDGFRKIGVMEKTDSRLMCAQCHVEYNCNAGS

QWSDGQKVGYDDQRTNHFPLKNAKDLLAHYKKLDFYDFKHAITGARLVKLQHPEAETYAGSVHDRAGVGCADCHMPRMKG

KDGKMFKSHGVIRPAHHVKEACLGCHPKSTVEQKSYQIEGTRNYIRGKMRKAEYWLGQLIDTYAAAKRMGIGEDVLAKAR

EKHEEAHVLWEYWTAENSDGFHNPELARDSLTSSIAASKAGVKLLNDAMEPKK

>OGP65189.1 cytochrome C [Deltaproteobacteria bacterium RBG_13_47_9]

MFATGAVGTLTLVLLLGLTAAATSKGAAKPVKDGKEQCYQCHDEVKALKEGSKHAAIPCGTCHGELAKHVESQSAVKALT

RIDASVCGKCHKDQFGSFHKVNYEAAARKEKGVPVGRSPMQDKLLAGHGFTFEHNEPRGHVFMLIDQFAVDRFQGGRFQF

REGWKGVDAIGKAWDVLIDTGKKLPETAMAGNPTCIQCKTSDHILTWKFMGDKGGKWDRTSHIVEMAKDTQNPMGCIHCH

DPHGAQPRVVRDALIQAISKDPAQNIFAKGGKTDLKEIEFQGFRKIGVMKKTDSRMMCAQCHVEYACNPGIQWSDGKRVG

YDDQRTNHFPLKNAKLLLEHYRKLDFYDFRHAVTGARLIKLQHPEAETYAGSVHDRAGVQCHQCHMPKQKGKDGRTFSNH

SMIRPKSHVKEACLGCHSKSTVEQKLYQIDAIQNYTKGKMRKAEYWLGQLIDSYATAKRAGVAEDILAKGQEKHEEAHVL

WEFWTAENSDGFHNPDLARDVLTASIASSKEGVVLLNRATVERTETVKSAEGKK

>WP_039647922.1 MULTISPECIES: cytochrome c [Geobacter] >KIE44032.1 cytochrome C [Geobacter soli] >ANA39291.1 cytochrome C [Geobacter anodireducens]

MWKKRLAVLAVAAGAAVVLSIPVLSTAAKGKPAATGAKGDGREACYGCHEEVKALKEGSKHARLSCESCHDNLKEHLANY

ENKPGTNLEPAKCGSCHKNEYDSFFTVNYEAQPRKEKGIPTGRSPMQDKLLAGHGFTFEHNEPRGHAFMVVDQFIVDRFQ

GGRFQYKKGAWGMDATGKTWDILTDTGAKLPETAMAGNPTCIQCKTSDHILTWKFMGDKDPKAKWDRSSDIVAVAKDTQN

PVGCIHCHDPHGTQPRVVRDGLIQAIEKDPSGNIFAKDGKTDLKVVSFRDGFRKIGVMEKTDSRLMCAQCHVEYNCNAGF

QWSDGQKVGYDDQRTNHFPLKNAKDLLAHYKKLDFYDFKHAITGARLVKLQHPEAETYAGSVHDRAGVGCADCHMPRMKG

KDGKTFKSHGVIRPAHHVKEACLGCHPKSTVEQKSYQIEGTRNYIRGKMRKAEYWLGQLIDTYAAAKRMGIGEDVLAKAR

EKHEEAHVLWEYWTAENSDGFHNPELARDSLTSSIAASKAGVKLLNDAMEPKK

>WP_010941025.1 cytochrome c [Geobacter sulfurreducens] >NP_951416.1 cytochrome c nitrite reductase [Geobacter sulfurreducens PCA] >AAR33689.1 cytochrome c nitrite reductase [Geobacter sulfurreducens PCA] >ADI83188.1 cytochrome c nitrite reductase, 8 heme-binding sites [Geobacter sulfurreducens KN400]

MWKKRLAVLAVAAGAAVALSIPALSTAAKGKPAATGAKGDGRETCYGCHEEVKALKEGSKHARLACDSCHDKLKEHLANY

ETKPGTNLDPAKCGSCHKNEYSSFFTVNYDAQPRKEKGIPTGRSPMQDKLLAGHGFTFEHNEPRGHAFMVVDQFIVDRFQ

GGRFQYKKGAWGMDATGKAWDILTDTGKKLPETAMAGNPTCIQCKTSDHILKWKFMGDKDPKATWDRSSDIVAVAKDTQN

PVGCIHCHDPHGTQPRVVRDGLIQAIEKDPTANIFAKNGKTDLKVISFRDGFRKIGVMEKTDSRLMCAQCHVEYNCNAGS

QWSDGQKVGYDDQRTNHFPLKNAKDLLAHYKKLDFYDFKHAITGARLVKLQHPEAETYAGSVHDRAGVGCADCHMPRMKG

KDGKMFKSHGVIRPAHHVKEACLGCHPKSTVEQKSYQIEGTRNYIRGKMRKAEYWLGQLIDTYAAAKRMGIGEDVLAKAR

EKHEEAHVLWEYWTAENSDGFHNPELARDSLTSSIAASKAGVKLLNDAMEPKK

>OGP33467.1 hypothetical protein A2X88_03755 [Deltaproteobacteria bacterium GWC2_65_14]

MKRTRALGFVVLLAAFTAWFAAGGTVAVKQAWGAPAKAKEEAPVQEAKCFDCHDGIEALKKGNRHGKVNCVSCHTGTAAH

LADSDKRPGTRFDHEACGACHKDQYETFTRLNMKRFARVEKSLLTERAPNPLWDKLMMGHGFTKEHAAPRSHKFMLVDHL

IVDRAYGGRFQSKTGWSYVAAPGNPKAWDVLEDRYPDSKEHKAFLPESAPAANPTCLQCKTQDQILKWKFLGDKDERAKW

DRTSNVVEFVKDLQNGLNCFFCHDPHAAKPRIVRDALIQALERPERDTLWHKDPRATKMKVVEFREGFRKIALLEKYDAK

LQCGQCHVEYNCNPGFDPKTGEYSIQSSDRRSNHFPFKDVFQIYDHYNALGFRDFKHGLTGGLLWKAQHPEAETFWNSVH

DRVGVSCNDCHMPKVKNRQGKVFTSHWQTTPRHYLQETCLSAKCHPKLTPDQAIYEIESVKSFVKGKMRKAEFWLGRLID

GIVEARKAGVDEAAIREAREQHQKAHILWEWWTAENSDGFHNPEAARESLTRSVVESKKGIQILSEAMEKKTAAK

>WP_011940785.1 cytochrome c [Geobacter uraniireducens] >ABQ28148.1 Formate-dependent nitrite reductase periplasmic cytochrome c552 subunit-like protein [Geobacter uraniireducens Rf4]

MWKKNLVVLAAIGGAMLAIALPTSSTAAKAKPAAKTKWDGKTCYTCHDDIKALKEGTKHAKLGCDVCHDELKKHLDSGGE

VKPVTRIDQTVCGKCHKDQFESFYKVDYEGQARKEKGVPTGRSPMMDKLLAPYGFTYEHNEPRGHAFMVTDQFVVDRFAG

GRFQYKKRWWDVDAVGKTWNILEDKGPDFKMGETAKAGNPTCIQCKTSDHILKWKFLGDKDPRAKWDRTSDIIAVAKDTN

NIMGCIHCHDPHGAQPRIVRDELINEIDKGAKMFSGKDGKTDLKVISFREGFRKIGVMQKVDSRMMCAQCHVEYACGKGF

EFNSGKGIGYDDQRTNHMPLKQVNDLLTHYKRLNFYDFKHAVTGARLVKLQHPEAETYSGSVHDRNGIKCDDCHMPRMKD

KRGKLFTSHMVIKPRNHVKEACLRCHPDSTVEKKLYQIDAIQNYIKGKMRKSEYWLGQLIDTYAEAKRFGVDETTLAKAR

EKHEEAHVLWEWWTAENSDGFHNPDLARDSLAASITASKEAVALLNKAIEEKTKKP

>WP_039739996.1 cytochrome c [Geobacter pickeringii] >AJE02265.1 cytochrome C [Geobacter pickeringii]

MWKKRLAAVAVVAGAGLAVSIPAFSTAAKGKPAATVKDDGREVCYGCHDEIKAMKEGSKHAKLACGVCHDKLKEHLASYE

NKPVTTIDAALCGSCHKDEFESFFTVNYDSQPRKEKGVPTGRSPMMDKLLAPYGFTFEHNEPRGHAFMVTDQFVVDRFAG

GRFQYKKRWFGVDSVGKTWDILEDKGPDFKMAESAKAGNPTCIQCKTSDHILKWKFMGDKDPRAKWDRTSDIIAVAKDTQ

NVVGCIHCHDPHGTQPRVVRDALIQAIDEKGAKMFAGKDGTTDLKVISFRDGFRKIGVMKKSDSRMMCAQCHVNYNCGTG

YEFDTGKKVGYEDQRTNHIPLAGPKDLLEHSKKQNFYELRHAVTGARLVKPRHPEAETYFGSVHDKAGVTCADCHMPIMK

NKQGKSFKSHMVIKPKDHVKEACLRCHPKYTAEQKLYQIQGIQNYIRGKMRKSEYWLGQLIDTYATAKRWGVDEETLAKA

REKHEEAHVLWEWWTAENSDGFHNPDLARDTLTASIAKSKEAVALLNKAIEAKSGGARGAEPKKQ

>OGL40262.1 hypothetical protein A2043_06675 [Candidatus Schekmanbacteria bacterium GWA2_38_9] >OGL50300.1 hypothetical protein A3H37_00920 [Candidatus Schekmanbacteria bacterium RIFCSPLOWO2_02_FULL_38_14] >OGL55389.1 hypothetical protein A3G31_01080 [Candidatus Schekmanbacteria bacterium RIFCSPLOWO2_12_FULL_38_15]

MRLKRLFLSFGVGVLVLLLGSFIISAKNSSGPNFVKPETCFECHVEVQELWSEGKHKKTLNCVKCHDNLNEHIASPEEKK

PVTNLNPENCGSCHKNQFETFFKTNPKKGARVEKSLLSERSPNPFWDKLMMGHGFTKEHNAPRSHAYMLVDHLVVDRAYG

GRFQPKKGWEYTVLPGPLKAWDVLEDRHPETNEHKPFLPESAAAAYPTCLKCKTNDSILKWKFMGDKDPKAKWDRASNVV

EYAKDLNNAIACIHCHDPHATKPRIVRDGLIQALTRPEEDTLWHKDSKRTGIKVIDFRDFRKIAILDKYDPKLQCGQCHV

EYNCNAGINPKTGEKIPASDVRTNHFPFKDVFQIYDHYNKLEFRDFKHSLTGGLLFKAQHPEAEVFWNSKHDKAGASCND

CHMPKIKGKDGKVFTSHWQTSPRNYIKETCLKCHSKWTAEQANYSIDAVRNHIKGKMRKAEFWLSSLIDKIVEAKKLGID

ENVINEAKEQHQKAHILWEWWTAENSDGFHNPEQARESLARSVEESKKGIELITKAVSEKK

>KRT74970.1 nitrite reductase [Deltaproteobacteria bacterium CSP1-8]

MKRTRALRSIVLLAAFTSWLAAVSTVAVKEARGAPPESKKEAPVPVQEAKCFDCHDGIEALKKGSKHGKVNCVSCHTGTA

AHLADSGKKPGTRFDHEACGACHKDQYETFTRLNMKRFARVEKSLLTERSPNPLWDKLMMGHGFTKEHAAPRSHKFMLVD

HLIVDRAYGGRFQSKTGWSYVAAPGNPMAWDVLEDRYPDSKEHKAFLPESAPAANPTCLQCKTQDQILKWKFLGDKDERA

KWDRTSNVVEFVKDLQNGLNCFFCHDPHAAKPRIVRDALIQALERPEKDTLWHKDPRATKMKVVEFREGFRKIALLEKYD

AKLQCGQCHVEYNCNPGFDPKTGEYSIKSSDLRSNHFPFKDVFQIYDHYNALGFRDFKHGLTGGLLWKAQHPEAETFWNS

VHDRAGASCNDCHMPKVKNRQGKVFTSHWQTTPRHYLQETCLTAKCHPKLTPEQAIYEIESVKSFVRGKMRKAEFWLGSL

IDKIVEARKAGVDEAMIREAQEQHQKAHVLWEWWTAENSDGFHNPAAARESLTRSVVESRKGIQILSEGMAKKTAAK

>WP_015836589.1 cytochrome c [Geobacter sp. M21] >ACT17337.1 Nitrite reductase (cytochrome; ammonia-forming) [Geobacter sp. M21]

MLKRNLALTAAAAVAVGFLSLPALTTAAKSQPAAKVASDGRAKCYECHDEVKALKEGSKHAKLSCKVCHEKLDAHMDDPE

KNKPVTLIDQALCGKCHKEQYNSFYEDNYEAAARKEKGSPTGRSPLQDKLLAGHGFTFEHAEPRGHAFMVIDQFAVDRFQ

GGRFQFKEGWKGVTSTGKTWDVLTDTGKKLPESAMAGNPTCIQCKTSDHLLKWKFLGDKDPRAKWDRTSNIVDVAKDTHN

PVGCVHCHDPHGTQPRVVRDALIQAIQKDPKGNIFAKNGATDLKVIDFRDFRKIGVMQKTDSRMMCAQCHVEYNCNAGTQ

WSDGKKVGYDDLRTNHFPLKNSLQLLKHYQELNFFDFKHAVTGARLIKFQHPEAETYAGSVHDRAGVQCHDCHMPKKKGK

DGKMFSTHGVIRPKNHIKEACLGCHPKEGKDKKFYQLDAVQNYVKGKMRKSEYWLGELIDSYAAAQRAGVSPTVLDEARA

KHEEAHALWEYWTAENSDGFHNPELARESLTGSIAASKAGVKILNDAMAVAKKDEPKK

>OGU17099.1 cytochrome C [Geobacteraceae bacterium GWC2_53_11]

MLKKNLALAAAVVGTAGLLLSLPALTTAVNKPATKLANDGRAKCYECHDEVKALKEGSKHAKLACTVCHDKLDAHMSDPE

KNKPVTVIDQALCGKCHKSQFESFYKVNYDGGARKEKGTPTGRSPMQDKLLAPYGFTFEHNEPRGHAFMVIDQFAVDRFQ

GGRFQFKDGWKGVDKVGKTWDVLEDKGKDYKLKETAMAGNPTCIQCKTSDHILKWKFMGDKDPKAKWDRTSNIVDVAKDT

NNAVGCIHCHDPHGAQPRVVRDGLIQAIEKDTKNNIFSKGGTTDLKVVSFRDGFRKIGVMKKTDSRMMCAQCHVEYNCGA

GNQWSDGGKVGYNDQRTNHFPLKNALQLLKHYKDLDFFDFKHAVTGARLIKFQHPEAETYAGSVHDKAGVQCHQCHMPKV

KGKDGKMFSAHGVIKPKQNIKAACLGCHPDSTVEKKQYEMDSVINYTKGKMRKAEYWLGQLIDSYAAAQRMGVVEDVLKQ

AREKHEEAHVLWEYWTAENSDGFHNPGLARESLTGSIAASKAGVKILNDAMAVAKKDEAKK

>OGL49840.1 hypothetical protein A3C43_12020 [Candidatus Schekmanbacteria bacterium RIFCSPHIGHO2_02_FULL_38_11]

MRLKRLFLSFGVGVLVLLLGSFIISAKNSSGPNFVKPETCFECHVEVQELWSEGKHKKTLNCVKCHDNLNEHIASPEEKK

PVTNLNPENCGSCHKNQFETFFKTNPKKGARVEKSLLSERSPNPFWDKLMMGHGFTKEHNAPRSHAYMLVDHLVVDRAYG

GRFQPKKGWEYTVLPGPLKAWDVLEDRHPETNEHKPFLPESAAAAYPTCLKCKTNFSILKWKFMGDKDPKAKWDRASNVV

EYAKDLNNAIACIHCHDPHATKPRIVRDGLIQALTRPEEDTLWHKDSKRTGIKVIDFRDFRKIAILDKYDPKLQCGQCHV

EYNCNAGINPKTGEKIPASDVRTNHFPFKDVFQIYDHYNKLEFRDFKHSLTGGLLFKAQHPEAEVFWNSKHDKAGASCND

CHMPKIKGKDGKVFTSHWQTSPRNYIKETCLKCHSKWTAEQANYSIDAVRNHIKGKMRKAEFWLSSLIDKIVEAKKLGID

ENVINEAKEQHQKAHILWEWWTAENSDGFHNPEQARESLARSVEESKKGIELITKAVSEKK

>WP_041971764.1 cytochrome c [Geobacter sp. OR-1] >GAM09844.1 cytochrome c-552 [Geobacter sp. OR-1]

MLKKTVTALVIVCGTALCLGIPAQLTAAKAKPAGKTQGKEACYQCHDEIKTMKENSRHAKLACETCHDNLGKHLESMGDV

KPVTRIDAALCGKCHKDQYDSFFKMNYESGARKEKGVPAGRSPQQDKLLAGHGFTFEHNEPRGHAFMVTDQFIVDRFQGG

RYQYKDRWYGYDKPGKTWDVLVDNGEKTKLPETAMAGNPTCIQCKTSDHILKWKFLGDKDPRAKWDRTSNVNDVARDTNN

PVGCIHCHDPHGAQPRVVRDALIQAIGRDPGSNIFAKSGRTDMKEIDFRGFRKIGVMAKTDSRMMCAQCHVEYNCNTGSQ

WSDNKKVGYDDQRTNHMPLKNAKDLLAHYKKLDFYDFRHSVTGARLIKLQHPEAETYAGSVHDRAGVQCHQCHMPKLKGK

EGKQFSTHGVIRPKNHIKEACLGCHPKYTVEAKLYQIETIQNYTKGKMRKAEYWIGKLIDSYAVAKRSGVSEETLAKARE

KHEEAHVLWEFWTAENSDGFHNPDLARDTLTGAIKAAKDGVKLLEDTMESKK

>WP_015721120.1 cytochrome c [Geobacter sp. M18] >ADW14273.1 Nitrite reductase (cytochrome; ammonia-forming) [Geobacter sp. M18]

MLKKNLVVTAAVAGAIALLSIPALTTAAKTKPVQVANDGRAKCYDCHDEVKALKEGSKHAKLSCKVCHDKLDAHMSDPEK

NKPVTLIDQALCGKCHKEQYDSFYEDNYEAAARKEKGIPTGRSPLQDKLLAGHGFTFEHNEPRGHAFMVIDQFVVDRFQG

GRFQFKEGWRGVTSTGKTWDVLTDTGKKLPETAMAGNPTCIQCKSSDHLLKWKFMGDKDPKAKWDRTSNVVDVAKDTHNP

VGCVHCHDPHGTQPRVVRDALIQAIEKDPKANMFAKNGATDLKVIDFRGFRKIGVMSKTDSRMMCAQCHVEYNCNAGTQL

SDGKPVKYDDLRTNHFPLRNSLQLLKHYQELNFFDFKHAVTGARLIKFQHPEAETYAGSVHDRAGVQCHQCHMPKKKGKN

GKMFSSHGVIRPKNHIKEACMGCHPKEVKDKKAYQIDAVQNYVKGKMRKAEYWLGQLIDSYAAAQRLGVSPAVLDEARAK

HEEAHALWEYWTAENSDGFHNPELARESLTGSIAASKAGVKILNDAMTVAKKEEPKK

>OGQ99239.1 hypothetical protein A2505_05840 [Deltaproteobacteria bacterium RIFOXYD12_FULL_55_16]

MHKKTLVTVAALFVLVPMAVVFAPKADAQAKKDGAVKAVQEKNVNAAACYECHGTVKELHLMGKHSKVNCVSCHDGLARH

LANPGKETRPVTRMSWETCGACHKDQYESFMKETFHRPARDEKSQLTNRSPNPYWDKLMAGHGFTKEHNLTRSHVNMLTD

QLAVDRSFGGRFQPKNGWNYIFEKGKTWDVLVDTHPETKEHKAFRPQTAAAANPVCLQCKTQDQILNWAYMGDKVEGAKW

SRQSNVVEVAKDVEHGLSCFTCHDPHAAKPRIVRDGLIDALTRPDGDTLWHKDPKRTGIKVIDMGTRGFTRKIALLDKYD

SRLQCGQCHVEYNCNPGTDTKTGQPVKMDDRRTNHFPYKDVFGLYDHYVNKINFLDFKHAITGGLLWKAQHPESESYYNS

KHAKAGVGCDACHTPKMKNKAGKVYTSHFAVTPRVMLKETCLKCHPQWTEEQAKYSIDSIKAHIKGKMRKAEYWLSALID

KIVEAKKAGVEEATIKKAQDQHLKAHILWEYWTAENSDGFHNPEMAKESLAKSMDESQAGIKLLTEAMAPKPAAAK

>OGR30974.1 cytochrome C, partial [Desulfuromonadales bacterium GWD2_54_10]

MLKKNLAFAAAIIGMLAVLSLPGLTTAAKSKPAAKPVNDGRATCYECHDEVKALKEGSKHAKLSCKTCHDKLDAHISDPE

KNKPVTIIDQALCGKCHKNQYDSFFNVNHDGGARKEKGVPTGRSPMQDKLLAPYGFTFEHNEPRGHAFMVIDQFVVDRFQ

GGRFQFKDGWRGIDKTGKTWDVLTDKGKDFKLKETAMAGNPTCIQCKTSDHILNMKFMGDKDPKAKWSRESNIVEVAKDT

NNPVGCIHCHDPHGTQPRIVRDGLLLAIAKDTKGNIFAKGGKTDLKEISFRDGFRKIGVMEKTDSRMMCAQCHVEYNCGA

GTQWSDAKKVGYDDQRTNHFPLKNALQLLKHYKDLDFFDFKHAVTGARLVKFQHPEAETYAGSVHDRAGVQCHQCHMPKM

KGKDGKMFSTHGVVKPKLTVKASCLGCHPDSTVEKKQYEMESIINYTKGKMRKAEYWLGQLIDTYAAAQRMGVTDSVLAQ

AREKHEEAHVLWEYWTAENSDGFHNPDLARESLTSSIAVSKAGVKILNDA

>OGR03770.1 hypothetical protein A2520_03510 [Deltaproteobacteria bacterium RIFOXYD12_FULL_53_23]

MRKKTVVTAMAMFALAPMVVMFAGNTEAEAKKAAIVKPAQEKPVNAAACYECHGTVKELHLMGKHSKVNCVSCHGGLAQH

LANPGKETRPETRMSWETCGSCHKDQYESFMKEAYHRPARDEKSQLTGRSPNPYWDKLMAGHGFTKEHNLTRSHVNMLTD

QLVVDRSFGGRFQPKNGWNYVLEKGKTWDILVDTHPETKEHKAFRPQTAAAANPVCLQCKTQDQILKWAYMGDKVEGVQW

SRQSNVVEVAKDVEHGLSCFTCHDPHAAKPRIVRDGLIEALTRPEGDTLWHKDPKRTGIKVIDMGVRGFTRKIALLDKYD

SRLQCGQCHVEYNCNPGTDTKTGKPVKMDDRRTNHFPFKDVFGLYDHYVNQINFLDFKHAITGGLLWKAQHPEAESYYNS

KHAKAGVGCDSCHTPKMKNKAGKVYTSHFAATPKVMLKETCLKCHPQWTEEQAKYSIDSIKAHIRGKLRKAEFHLSNLID

KIVEAKKAGVAEETIKKAQDQHLKAHILWEYWTAENSDGFHNPEMAKEALGKSMNESLAGIKLLTEAMAPKAAAK

>WP_012531374.1 cytochrome c [Geobacter bemidjiensis] >ACH39949.1 cytochrome c nitrite reductase [Geobacter bemidjiensis Bem]

MLKRNLALTAAAAVAAGFLSLPALTTAAKSNPAAKVASDGRAKCYECHDEVKALKEGSKHAKLSCKVCHEKLDAHMNDPE

KNKPVTLIDQALCGKCHKEQYNSFYEDNYEAAARKEKGTPTGRSPLQDKLLAGHGFTFEHDEPRGHAFMVIDQFAVDRFQ

GGRFQLKEGWKGITSTGKTWDVLTDTGKKLPETAMAGNATCIQCKTSDHLLKWKFLGDKDPRAKWDRTSNVVDVAKDTHN

PVGCVHCHDPHGTQPRVVRDALIQAIQKDPKGNIFAKNGATDLKVIDFRDFRKIGVMQKTDSRMMCAQCHVEYNCNAGTQ

WSDGKKVGYDDLRTNHFPLKNSLQLLKHYQELNFFDFKHAVTGARLIKFQHPEAETYAGSVHDKAGVQCHQCHMPKKKGK

DGKMFSTHGVIRPKNHIKEACLGCHPKEGKDKKLYQLDAVQNYVKGKMRKSEYWLGQLIDTYAAAQRAGVSPTVLDEARL

KHEEAHALWEYWTAENSDGFHNPELARESLTGSIAASKAGVKILNDAMSVAKKDEAKK

>WP_026842615.1 cytochrome c [Geobacter bremensis]

MLKRNLALTAAAAVAAGFLSLPALTTAAKSNPAAKVASDGRAKCYECHDEVKALKEGSKHAKLSCKVCHEKLDAHMNDPE

KNKPVTLIDQALCGKCHKEQYNSFYEDNYEAAARKEKGTPTGRSPLQDKLLAGHGFTFEHDEPRGHAFMVIDQFAVDRFQ

GGRFQLKEGWKGVTSTGKTWDVLTDTGKKLPETAMAGNATCIQCKTSDHLLKWKFLGDKDPKAKWDRTSNVVDVAKDTHN

PVGCVHCHDPHGTQPRVVRDALIQAIQKDPKGNIFAKNGATDLKVIDFRDFRKIGVMQKTDSRMMCAQCHVEYNCNAGTQ

WSDGKKVGYDDLRTNHFPLKNSLQLLKHYQELNFYDFKHAVTGARLIKFQHPEAETYAGSIHDKAGVQCHQCHMPKKKGK

DGKMFSTHGVIRPKNHIKEACLGCHPKEGKDKKLYQLDAVQNYVKGKMRKSEYWLGQLIDTYAAAQRAGVSPTVLDEARL

KHEEAHALWEYWTAENSDGFHNPELARESLTGSIAASKAGVKILNDAMSVAKKDEPKK

>OGU02519.1 cytochrome C [Geobacteraceae bacterium GWC2_55_20] >OGU24670.1 cytochrome C [Geobacteraceae bacterium GWF2_54_21]

MLKKNLAFAAAIAGAAALLSLPAMTTAAKSKPAAKVADDGRAKCYECHEEVKALKEGSKHAKLSCRTCHDKMDAHMQDPE

KNKPVTIIDQALCGKCHKNQMESFYNVNRDGGARKEKGVPTGRSPMQDKLLAPYGFTFEHNEPRGHAFMVIDQFAVDRFQ

GGRFQFKDGWKGIDKTGKTWDVLTDKGKDYKLKETAMAGNPTCIQCKTSDHILKWKFMGDKDPKAKWDRTSNIVDVAKDT

NNPVGCIHCHDPHGTQPRVVRDGLIQAIERDTKGNIFAKGGKTDLKVISFRDGFRKIGVMEKTDSRMMCAQCHVEYACGA

GTQWSDGKVVKYDDQRTNHFPLKNALQLLKHYKELDFFDFKHAVTGARLVKFQHPEAETYAGSVHDRAGVQCHQCHMPKI

KGKDGKMFSTHGVVKPKLAVKASCLGCHPDSTVEKKQYEMESIINYTKGKMRKAEYWLGQLIDSYAAAQRMGVTESVLAQ

AREKHEEAHVLWEYWTAENSDGFHNPDLARESLTSSIAVSKAGVKILNDAMAVVKKDEVKK

>OGQ88307.1 hypothetical protein A2512_03100 [Deltaproteobacteria bacterium RIFOXYD12_FULL_56_24]

MYKKTVVTAAALFVLAPMAVMFVPEADAQAKKGGVAKAVQEKNVNPAACYECHGTVKELHTMGKHSKVNCVSCHGGLANH

LANPGKDTRPETRMSWETCGACHKDQYDSFMKQAYHRPARDEKSQLTNRSPNPYWDKLMAGHGFTKEHNLTRSHVNMLTD

QLAVDRSFGGRFQPKNGWNYIFEKGKTWDVLVDTHPETKEHKAFIPQSAAAANPVCLQCKTQDQILKWAYMGDKVEGAQW

SRQSNVVEVAKDVEHGLSCFTCHDPHAAKPRIVRDGLIDALTRPDGDTLWHKDPKRTGIKVIDMGVRGFPRKIALLDKYD

SRLQCGQCHVEYNCNPGTDTKTGQKVTMADRRTNHFPYKDVFGLYDHYVNKINFMDFKHAITGGLLWKAQHPESESYYNS

KHAKAGVGCDSCHTPKLKNKAGKVYTSHFAVTPRVMLKETCLKCHPQWTEEQAKYSIDSIKAHIKGKMRKAEYHLSNLID

KIVEAKKAGVDEATIKKAQDQHLKAHILWEYWTAENSDGFHNPEMAKEALTKSVNESMAGIKLLTEAMTPKTAAAK

>WP_012648182.1 cytochrome c [Geobacter daltonii] >ACM21454.1 cytochrome c nitrite reductase, 8 heme-binding sites [Geobacter daltonii FRC-32]

MINRRLASVAAATGMAMLLSLPIYSTAAKGKPAAKDDGRETCYGCHDQVKALKEGSKHAKLACSTCHDKLKEHLDNSETR

PVTLIDSALCGKCHKDEYATSMMVDYEAPARKEKGIPGGRSPVMDKLLAPYGFTIEHNEPRGHAFMVTDQFVVDRFAGGR

FQYKQRWAGVDQVGKTWDILEDKGPDFKMGETGKAGNPTCIQCKTSDHILKWKFLGDKDPKAKWDRTSDIIAVAKDTHNP

VGCIHCHDPHGAQPRIVRDALINEIDKGATMFARNGATDLKVISFRDGFRKIGVMKKSDSRMMCAQCHVEYACGKGFEFG

SGKPVGYDDQRTNHMPLKQVKELLDHYRKLNYYDFKHAVTGARLVKLQHPEAESYAGSVHEKAGVTCADCHMPVMKNKQG

KSYKSHMMIRPRSHVKESCQGCHPKWNAEQQLYQIDAIRNYISGKMRKSEYWLGELIDTYAAAKRFGVDEATLAKAREKH

EEAHVLWEWWTAENSDGFHNPDLARDSLAASITASKEGVALLNKALDERGKK

>OHE55935.1 hypothetical protein A2Z47_13875 [Thermodesulfovibrio sp. RBG_19FT_COMBO_42_12]

MFKKSLILFAAIAVLFSLSTVFSTRAEKGSVIKSQTCFACHPEMAKLWDKGKHKSAKIECTQCHSELAAHLASNPPGKMK

PVTNLNPSNCGGCHRDQFETFFTVDYTESARSEKSLFTERSPNPLWDKLMMGHGFTKEHNAPRGHAYMLLDHLVVDRAYG

GRFQPKEGWQYIVAPTPVSLWDVLEDRYPASKEHKAFIHESAPAANPTCLQCKTTDNILKWKHLGEKDPAAKWDRTSNVV

ELVKDIQNPVGCIHCHDPHATGPRIVRDALIQALTRPEADTLWHKDSKRTEIEVIDFRNFRKTALLNKYDTKLLCGQCHV

EYNCNPGVSAKTGEKIGFNDLRTNHFPMKDVFQIYDHYNQADFRDFKHTLTGGLLFKAQHPEAEVFWNSKHDKAGVGCND

CHMPKIKNKAGKVFTSHWQTSPKKYIKETCLKCHKEWTEEMAVYTIDSVKNHVQGKMRKAEFWLDQLIDKIVTAKKAGID

EAVIKDAQEQHQKAHILWEWWTAENSDGFHNPDQARESLTRSVEESKKGIELLTKAISEKK

>WP_012469134.1 cytochrome c [Geobacter lovleyi] >ACD94784.1 cytochrome c family protein [Geobacter lovleyi SZ]

MLKQRLAVAASIVGLGALLAIPSLAAPKAKPAAKPANDGREQCYMCHTEVKALKEASKHAALACSVCHDKTKEHLANPGP

DTKPVTKLDPAVCGSCHKNQYQSFFTPHYPEGGSRKEKGVPTGRSPMQDKLLAGHGFTFEHAEPRGHAFMVVDQFAVDRF

QGGRYQFKGGWKNYDKVGKTWDVLEDKGASAKLTETAMAGNPTCIQCKTSDLILKWKYLGEKGGKFDRTSNVNDVMKDVH

NPVGCIECHDPHGTQPRVIRDALIAAIDKDPAKNIFAKNGKTDLKVVSFRDGFRKIGVMEKTDSRMMCAQCHVEYNCNPG

TQWSDGKPVTFASDRTNHFPLKNAKEILAHYKQLDFFDFKHAVTGARLIKLQHPEAETYAGSVHDKAGVQCHQCHMPKLK

GKDGKPYSTHGVVKPINMAKESCISCHKDSSVEKAKWQIDTIRNYTKGKMRKAEYWLGQLIDTYLVASRAGVAPAVLDQA

RAKHEEAHVLWEYWTAENSDGFHNPALARETLTGSIAASKAGVKILNDAMAVANKKEEPKK

>OGW45057.1 hypothetical protein A2078_10520 [Nitrospirae bacterium GWC2_57_9]

MKRSVLSVGAAAAAVVLMIGLAGLTADAQYKKAAGAKAAGEKDVQVKTCYECHDPIQQLHTMGRHGKVNCVNCHSGLAKH

VANPGPDTRPGTDTSWEACGKCHKDQYDSFMQVAKHRPARDEKSQLTNRSPNPFWDKLMAGHGFTKEHSLTRSHVNMLVD

QFVVDRAFGGRFQPKNGWQYVNEKGKVWDVLTDTHQETKEHKAFMTQSAAAANPTCLQCKTQDHILKWSYLGEPVPGATW

SRISNVVEVAKDVNHGLNCFVCHDPHAAKPRVVRDGLIAALTRPEADTLWHKDPRHTNIKVIDMGTRGFTRKIAILEKYD

TKLQCGQCHVEYNCNDGYDPRNPDTSKKTVGYNSPLTNHFPYKDVFGLYDHYVKQVNFLDFKHAITGGLLWKAQHPEAEA

FYNSKHAKAGAGCDSCHTPKMKNKQGKVFTSHFAVTPKEHIKESCLTAKCHPGWTKQDAVYAIDSVKAHIRGKMRKAEFR

LSALIDKIVAAKAAGVDSEVIKKAQDQHLKAHILWEFWTAENSDGFHNPDMAKEALLKSADEAFKGIKMIDDAMAPKTAS

AAPAR

>OGW35682.1 hypothetical protein A2010_06725 [Nitrospirae bacterium GWD2_57_9]

MKRSVLSVGAAAAAVVLMIGLAGLTADAQYKKAAGAKAAGEKDVQVKTCYECHDPIQQLHTMGRHGKVNCVNCHSGLAKH

VANPGPDTRPGTDTSWEACGKCHKDQYDSFMQVAKHRPARDEKSQLTNRSPNPFWDKLMAGHGFTKEHSLTRSHVNMLVD

QFVVDRAFGGRFQPKNGWQYVNEKGKVWDVLTDTHQETKEHKAFMTQSAAAANPTCLQCKTQDHILKWSYLGEPVPGATW

SRISNVVEVAKDVNHGLNCFVCHDPHAAKPRVVRDGLIAALTRPEADTLWHKDPRHTNIKVIDMGTRGFTRKIAILEKYD

TKLQCGQCHVEYNCNDGYDPRNPDTSKKTVGYNSPLTNHFPYKDVFGLYDHYVKQVNFLDFKHAITGGLLWKAQHPEAEA

FYNSKHAKAGAGCDSCHTPKMKNKQGKVFTSHFAVTPKEHIKESCLTAKCHPGWTKQDAVYAIDSVKAHIRGKMRKAEFR

LSALIDKIVAAKAAGVDAEVIKKAQDQHLKAHILWEFWTAENSDGFHNPDMAKEALLKSADEAFKGIKMIDDAMAPKTAS

AAPAR

>OGR32206.1 cytochrome C [Desulfuromonadales bacterium GWC2_61_20] >OGR36045.1 cytochrome C [Desulfuromonadales bacterium GWD2_61_12]

MKQAGRLWSGCTVLTLFFLAVVGADAAPAAKGVSPADCFACHEEVKELHQGSKHGKLACETCHSGLAEHLQSEANKPLTN

LELSVCGNCHQDQFASFYRVNWDAQARKEKGTPTGRSPFQDKLLAPHGFTKEHNEPRAHAFMVVDQLTVDRFAAGRFQFK

DLFGYTRPGKTWDVLVDTEKTFPKDVSAAAGNPVCLQCKTSDLSLKWKYMGDKDTKAKWDRTSDVNALVKDVNNPMGCIM

CHDPHAAKLRITRDALIDAVEREGGTRPYDADKGAATVKIQVVSFRDGFRKIGLLDKPNSTLQCGQCHVEYNCNGGFEPE

SGDKVTMADRRANHFPLKNALDLLAHYDQLKFRDFKHAVTGARLIKLQHPEMETYWGSVHDKAGVTCGDCHMPKEKNKAG

KTFTSHQVVRPKDHVKASCVNCHAHKDSSVEEKLYQIQTVQNYTRGRMREAEVAIAKLIDTYAKAKEKGIAEETLAQARK

QHEIAHVLWEWWTAENSDGWHNPKLAQDSLFTAIVEANKGSELLLTAMAPPAPAAAK

>WP_035055218.1 cytochrome c [Desulfuromonas sp. TF]

MRGWWKGMLPLLTGAALVSMAALSAAQAPNGEKVDAAGCFGCHEEVQTLWKGSSHVDLGCDTCHAGMPAHLKNPQEKPRT

DLNPEVCGACHQNQYETFFQVNWNSPARQEKGVPEGRSPQQDKLLAPHGFTVEHNEPRSHPFMVADMLVVDRFSAGRYQF

KERWGVTRPGKVWDVVEDTGRTLPATTKAGNPVCLQCKTSDLILKWKFLGDKDPGAKWDRTSDVDAMVKDVQNVMGCIHC

HDPHAARSRIVRDALIEAVSREGINPYAPDPQKNAVEVVTFRDFRRIGLINEGGAGLVCGQCHVEYSCNPGITPEGEKIG

FDDRRTNHFPWRNALDILEQYDTIGFRDFRHAVTGARLVKLQHPEFETFWGSVHERAGVKCIDCHMVPAKNGAGETFTSH

LMVGPRERIEQVCLRCHPESTAEEALYQVDAVQNYTRGKMRKAEYHLELLIDTFEEAARQGVPEEALAAARKQHEIAHVL

WEWWTAENSDGWHNPSLARESLAASEAAAKEGIKTLQDAMKAKTGAPVVR

>WP_049770114.1 hypothetical protein [Calditerrivibrio nitroreducens]

MRRFFLLVLSFMLVSFGSVVFAAKTAPKQAAPDYAKVVKKGVDYNKCYECHDTIKELRGMGKHSNLACENCHGNIDAHLK

DSTNHPTVYTEWQACGKCHKEQFESFMTVSKHRPARDEKSQITNRAPNPYWDKLMMGHAFTKEHALTRSHPFMLLDQVLV

DRAAGGRFQPKNGWMYVNEKPQKIWDVIVDTQPGSEHKAFMKQTAAAVNPVCFQCKTQDHILDWAYLGDPNTSAPFNRKS

NPTAMIQSKKMQHGLNCYQCHDPHAAKPRIVRDALIAALTRPEADTLWHKDPSHTNFKVIDMGMRGYTRKIAILEKYDSR

LLCGQCHVEYVCNPGEDRNTGAKIGYDSPLTNHFPYKDVLQLYDHYVNQLHFTDFIHPLTGAKLVKFQHPESETFYNSKH

AKAGAGCDSCHTPKVKDKKTGKVYTSHYAVTPKHDLKASCLTSKCHSNWTEEDAKYAIDSVKAYIKGKMRKAEFWLDRLI

DKIVEGKNADLPADVIAKAQDYHVKAHILWEYWTAENSDGFHNPELARESLAQSIDASMAGVKLIDDALKAKKASAQQTQ

AQK

>ADR18397.1 nitrite reductase (cytochrome; ammonia-forming) [Calditerrivibrio nitroreducens DSM 19672]

MMRRFFLLVLSFMLVSFGSVVFAAKTAPKQAAPDYAKVVKKGVDYNKCYECHDTIKELRGMGKHSNLACENCHGNIDAHL

KDSTNHPTVYTEWQACGKCHKEQFESFMTVSKHRPARDEKSQITNRAPNPYWDKLMMGHAFTKEHALTRSHPFMLLDQVL

VDRAAGGRFQPKNGWMYVNEKPQKIWDVIVDTQPGSEHKAFMKQTAAAVNPVCFQCKTQDHILDWAYLGDPNTSAPFNRK

SNPTAMIQSKKMQHGLNCYQCHDPHAAKPRIVRDALIAALTRPEADTLWHKDPSHTNFKVIDMGMRGYTRKIAILEKYDS

RLLCGQCHVEYVCNPGEDRNTGAKIGYDSPLTNHFPYKDVLQLYDHYVNQLHFTDFIHPLTGAKLVKFQHPESETFYNSK

HAKAGAGCDSCHTPKVKDKKTGKVYTSHYAVTPKHDLKASCLTSKCHSNWTEEDAKYAIDSVKAYIKGKMRKAEFWLDRL

IDKIVEGKNADLPADVIAKAQDYHVKAHILWEYWTAENSDGFHNPELARESLAQSIDASMAGVKLIDDALKAKKASAQQT

QAQK

>WP_023274928.1 hypothetical protein [Mucispirillum schaedleri] >ESJ99779.1 hypothetical protein N508_00078 [Mucispirillum schaedleri ASF457]

MKKILKYSVMAFITALLFVFAAACQNNNSTSASAQAPASSADNAVVKAGYDRGALAASKADFTLAAGKVKENAGKTDKET

CFSCHTGVSELHNRGLHKDLDCSNCHFDITTEHTEAPSPANRPKVNMNWEACGQCHDNQMHSFLDVGKHRPARFEKSNFN

GRSPNPAWDKLMAPYGFTKEHAATRSHSLMLIDQFVVDRAFGGQFQPKDGWNYIFESGPVWDVLYDAKEKDPDFKDLPQT

ARAVNPVCMNCKTMDHMLDWAYLGEPNPKAKWSRLSNPVEMAKNMNHALNCFFCHDPHSAEPRIVRDALIEAISSDAPYA

KNNLYQQDANKVKAEIRVIDTRSMMDDTEPKMIRKIAILPKDDPRRQSLQCAQCHVEYNCGAGTDLGTGAKVGMNDPRTN

HFPLKNALALYDHYFVNLKFADFKNKFSGAMLWKGQHPEYETYYNSVHDKAGISCVQCHMETVEKGGKLDYTSHFVQSPR

YILNATCLTSDCHGTGADKHANWQGKDAEYVKVSTNWTAEDAKYSIDSIKTYTTGKMRKAEFWLAQLIDTIALAERLGVD

KATLDAARTQHSKAHILWEYWTAENSDGFHNPELARESLTQSINESMKGYDMLDAAMKK

>WP_005432949.1 MULTISPECIES: cytochrome c [Sutterella] >EKB32358.1 hypothetical protein HMPREF9465_00041 [Sutterella wadsworthensis 2_1_59BFAA] >KXT39414.1 cytochrome C family protein [Sutterella sp. KLE1602]

MHKQLTPVAAALALLGLLAGAPAQAADNQCAACHANVAKDHAGAAHKDLACTTCHTGTEAHLKDMKKRPAVNMDPAQCGA

CHQQQYKSAFMTSDRPARQSKKAAGGPAPDPFFDRALGGHGFTKEHDLPRAHTFMAIDQFIVDRAFGGRFEPKDGWLYTT

LEGGKSYKAWDVLKDNYPDNNEQKAHKPGTAAAANGVCWSCKSTDLMLDWAYMGDKAEGAKFHRGSNPVDVVRNVQHGMN

CNFCHDPHSAKPRIMRDALIDAMTREGKMNVYKDFAARQAKLEVKDVGVRGFDRKVAMLDRADSRLMCAQCHVEYVCNPG

MNVKTGEKVGFDSRLTNLFPWVNADQIEAYYDEVGFRDFKHNLTGATLVKMQHPDAETYFGSKHDKAGADCASCHMPKVK

DENGKTYTMHWATSPKHYVKETCLSCHKDKNEKQMVAAIDAMKGHFDGKVREAESRMNDMFNAFELAKTVGVSEEVLAKA

RKLHESAHINWEYWTAANGAYFHNHDMAVRSLAKSAKAASDATALLRKAIDEKAATKK

>CDD71990.1 cytochrome c family protein [Sutterella sp. CAG:397]

MIKHVMQACLAAAVALAIAPNASAAEKVNTTMCVACHSNVGDFHKSGAHKSVSCTSCHTGLAKHLKAPGKDTRPVTSMDP

KTCGSCHPAQFKSMYTVNDHRTPRASKKAAEGVAPDPFFDRALGGHGFTKEHAELRSHTFAVLDQFLCDRAFGGRFVPKD

GWLYLSMGSGAIDAWKVMNDLYPKDNLQKPRRPGTAAAANPVCISCKSTDLMLDWAYMGDKVEGATFSRESNVVDMARKT

SHSVNCNFCHDPHSAKPRVVRDGLIQALTRTDFPTVYSEDPNRTKIEVKDMGVRGFTRKIAILEKADSKLMCAQCHVEYN

CNPGIDTKTGKPVKMSDQRTNVFPFVAVDRIDDFYKHINFKDFKHNTTGALLTKLQHPDVETYWTSKHGKAGVDCKDCHM

PKVKGKDGKTYTNHWATSSKYYVKETCLRCHKDKTEAQMKRTISGMHAYYMGKLREAESRMTDMFNAFDLANAMGVDEAA

LKKARELHSVAHTNWEYWTAVNGAWFHNPEMAQASLAKSAAAAQEATKVLRDAMAAKRAK

>WP_016474744.1 hypothetical protein [Sutterella wadsworthensis] >EPD98506.1 hypothetical protein HMPREF1476_01545 [Sutterella wadsworthensis HGA0223]

MNTKPMLTAVAAALFSLGACVSAQAADLSTCTTCHSNITKAHAGAAHKDIACTSCHSGVDQHLKKVTDRPTVNMDPATCG

GCHQAQYKSLYKDDGRAARQSKKAPNGPAPDPFFDRALGAHGFTVEHDLPRAHVWMAIDQFIVDRAFGGRFEPKDGWLYA

TLDGGKSYKVWDVLKDNYPDNNAQKVHKPGTAAAANAVCWSCKSTDVMMDWAYLGDKVEGAQFSRSSNPVDVVRKVNHAI

NCNFCHDPHTAQPRVVRDALIDAVTRDDKSVPNVYRDVAAHPTKLDVKDVGVRGFTRKIAYMDKADSNLMCAQCHVEYIC

NPGFDAKTGAKIGFDSRLTNHFPFVNADEIEQYYDKIGFRDFKHNLTGAALVKMQHPDTETYFGSTHDKAGATCATCHMP

KVKDEKTGKMYTVHWATSPRHYMQETCLTCHKDKTAEQMNKAIDAMKGHYDGKVREAEARMNDMFNAFELAIASGVDEKT

LDEARKLHSSAHINWEYWTAVNGAYFHNPEEAQRSLAKSAKAASDATALLRKAIAAKASAKAGK

>WP_066593334.1 cytochrome C [Burkholderiales bacterium YL45] >ANU66139.1 cytochrome C [Burkholderiales bacterium YL45]

MRKMNNLLSILAVLALSGTASAALAQAPAQPNAASATCVACHSNIGAMHQGNKHQNLPCATCHEGTDKHLTNPKQKPTVS

MNPQQCGTCHADQFKTMYQANPHSIARNSKKNSDSIAPNPFFDEALGAHGFTKEHNLLRAHAYGAVDQFLADRSFGGRFV

LKDGWLFFGENGGFFGVWDKIKDTMPDNNQQKPHKPGTAAAANPVCWTCKSSDLMMDWAYLGDPKAGAKWSRASNPVELV

RNINHALNCNLCHDPHSAQPRVIRDALIQAVTRTDYPTLYSESANKTPVDVKDMGLRGFTRKIAILGKPDSKLMCAQCHV

EYNCNPGTDPATGKPIKMDDARTNLFPLVDVTKIDDFYKHASFKDFKHNQTGALLTKMQHPDTEIYWNSTHDKAGVGCAA

CHMPKVKNEKGETYTSHWATNPRAYVQETCLQCHKDKTEEHMTRVMDAQKAYYMGKLRETEGAMSQMFIAFREAKDAGIT

GEPIKQAQDLHSVAHTNWEWWTAANGAWFHNPQQAQASLAKATDSAKAATKILRDAVAAKVQGQQQAKK

>WP_005429704.1 cytochrome c [Sutterella wadsworthensis] >EFW02376.1 hypothetical protein HMPREF9464_00564 [Sutterella wadsworthensis 3_1_45B]

MLTAVAAALFSLGACVSAQAADLSTCTTCHSNITKAHAGAAHKDIACTSCHSGVDQHLKKVTDRPTVNMDPATCGGCHQA

QYKSLYKDDGRAARQSKKAPNGPAPDPFFDRALGAHGFTVEHDLPRAHVWMAIDQFIVDRAFGGRFEPKDGWLYATLDGG

KSYKVWDVLKDNYPDNNVQKAHKPGTAAAANAVCWSCKSTDVMMDWAYLGDKVEGAQFSRSSNPVDVVRKVNHAINCNFC

HDPHTAQPRVVRDALIDAVTRDDKSVPNVYRDVAAHPTKLDVKDVGVRGFTRKVAYMDKADSNLMCAQCHVEYICNPGFD

AKTGAKIGFDSRLTNHFPFVNADEIEQYYDKIGFRDFKHNLTGAALVKMQHPDTETYFGSTHDKAGATCATCHMPKVKDE

KTGKMYTVHWATSPRHYMQETCLTCHKDKTAEQMNKAIDAMKGHYDGKVREAEARMNDMFNAFELAIASGVDEKTLDEAR

KLHSSAHINWEYWTAVNGAYFHNPEEAQRSLAKSAKAASDATALLRKAIAAKASAKAGK

>CCZ16731.1 putative uncharacterized protein [Sutterella wadsworthensis CAG:135]

MNTKPMLTAVAAALFSLGACVSAQAADLSTCTTCHSNITKAHAGAAHKDIACTSCHSGVDQHLKKVTDRPTVNMDPATCG

GCHQAQYKSLYKDDGRAARQSKKAPNGPAPDPFFDRALGAHGFTVEHDLPRAHVWMAIDQFIVDRAFGGRFEPKDGWLYA

TLDGGKSYKVWDVLKDNYPDNNAQKVHKPGTAAAANAVCWSCKSTDVMMDWAYLGDKVEGAQFSRSSNPVDVVRKVNHAI

NCNFCHDPHTAQPRVVRDALIDAVTRDDKSVPNVYRDVAAHPTKLDVKDVGVRGFTRKVAYMDKADSNLMCAQCHVEYIC

NPGFDAKTGAKIGFDSRLTNHFPFVNADEIEQYYDKIGFRDFKHNLTGAALVKMQHPDTETYFGSTHDKAGATCATCHMP

KVKDEKTGKMYTVHWATSPRHYMQETCLTCHKDKTAEQMNKAIDAMKGHYDGKVREAEARMNDMFNAFELAIASGVDEKT

LDEARKLHSSAHINWEYWTAVNGAYFHNPEEAQRSLAKSAKAASDATALLRKAIAAKASAKAGK

>WP_008810882.1 cytochrome c [Parasutterella excrementihominis] >EFL83481.1 putative nitrite reductase [Burkholderiales bacterium 1_1_47]

MTKMPLFPSVLALALIGYFGVSYAAPAQNDANCTACHSNIGTLHTGSKHANLPCATCHDGTADHLKNPKTHPTVSMSPQQ

CSSCHPNQFETMYKVNDHRIARDSKKNLNNISPNPFFDEALGAHGFVKEHNLPRSHAYAAVDQFIADRSFGGRFQPKEGW

LFSGDNGGFFGVWEKIDDTIPGNAQKPHKPGTAAAANPVCWTCKSTDVMMDWAYLGDPNTKAKWSRASNPVDLVHNINHA

LNCNMCHDPHSAQPRIVRDALIQAMTRTDYPTLYSESANKTPIEVKDMGLRGFTRKIAILGKPDSKLMCAQCHVEYNCNP

GTDPATGKPIKMDDPRTNLFPLVDVTRIEDFYKHASFKDFKHNQTGALLTKMQHPDTEVYWNSKHDQMGVGCAACHMPKV

KDANGDVYTSHWATTPRAYIQETCLQCHKDKTEAQMNKVLDSMNAHYNGKLREAEASMGQMFIAFRQANDAGVDPAVIKQ

AQDLHSVAHTNWEWWTASNGSWFHNMPQAKESLAKSVAASQKATKLLRDAVAAKVAAQAQPQAQQTAQK

>CDA42889.1 nitrite reductase [Proteobacteria bacterium CAG:139]

MTKMPLFPSVLALALIGYFGASYAAPAQNDANCTACHSNIGTLHTGSKHANLPCATCHDGTADHLKNPKTHPTVSMSPQQ

CSSCHPNQFETMYKVNDHRIARDSKKNLNNISPNPFFDEALGAHGFVKEHNLPRSHAYAAVDQFIADRSFGGRFQPKEGW

LFSGDNGGFFGVWEKIDDTIPGNAQKPHKPGTAAAANPVCWTCKSTDVMMDWAYLGDPNTKAKWSRASNPVDLVHNINHA

VNCNMCHDPHSAQPRIVRDALIQAMTRTDYPTLYSESANKTPIDVKDMGLRGFTRKIAILGKPDSKLMCAQCHVEYNCNP

GTDPATGKPIKMDDPRTNLFPLVDVTRIDDFYKHASFKDFKHNQTGALLTKMQHPDTEVYWNSKHDQMGVGCAACHMPKV

KDANGNVYTSHWATTPRAYIQETCLQCHKDKTEAQMNKVLDSMNAHYNGKLREAEASMGQMFIAFRQANDAGVDPAVIKQ

AQDLHSVAHTNWEWWTASNGSWFHNMPQAKESLAKSVAASQKATKLLRDAVAAKVAAQAQPQAQQTAQK

>EGG57570.1 nitrite reductase [Parasutterella excrementihominis YIT 11859]

MTKFSYGRPFPCFGNKTRNGYGIVLISEEVDKREIRRYTVFKVELRLIRLQTYIVNKYFGSHTPRKSFGLFKDLKMTKMP

LFPSVLALALIGYFGVSYAAPAQNDANCTACHSNIGTLHTGSKHANLPCATCHDGTADHLKNPKTHPTVSMSPQQCSSCH

PNQFETMYKVNDHRIARDSKKNLNNISPNPFFDEALGAHGFVKEHNLPRSHAYAAVDQFIADRSFGGRFQPKEGWLFSGD

NGGFFGVWEKIDDTIPGNAQKPHKPGTAAAANPVCWTCKSTDVMMDWAYLGDPNTKAKWSRASNPVDLVHNINHALNCNM

CHDPHSAQPRIVRDALIQAMTRTDYPTLYSESANKTPIEVKDMGLRGFTRKIAILGKPDSKLMCAQCHVEYNCNPGTDPA

TGKPIKMDDPRTNLFPLVDVTRIEDFYKHASFKDFKHNQTGALLTKMQHPDTEVYWNSKHDQMGVGCAACHMPKVKDANG

DVYTSHWATTPRAYIQETCLQCHKDKTEAQMNKVLDSMNAHYNGKLREAEASMGQMFIAFRQANDAGVDPAVIKQAQDLH

SVAHTNWEWWTASNGSWFHNMPQAKESLAKSVAASQKATKLLRDAVAAKVAAQAQPQAQQTAQK

>WP_008542152.1 cytochrome c [Sutterella parvirubra] >EHY31355.1 cytochrome C family protein [Sutterella parvirubra YIT 11816]

MHHQTKHLAALIAAIGLSAATGAWAAPDLSACQTCHADLTKTHASAGHKDVACTTCHGGVEDHLKNMKQRPTVSMDPATC

GACHQDQYKSLFTVSDRPARQSKKAAEGPAPDPFFDRAMGAHGFTKEHDLPRAHTFMAIDQFIVDRAFGGRFEPKEGWLY

TTLEGGKSYKVWDVLKDNHPENNEHKPFKPGTAAAGNAVCWTCKSSDLMLDWAYMGDKVEGAAFNRGSNAVDVVRKVNHA

INCNFCHDPHNTKPRIIRDALIESIERKDGPNVWHEVAAHKTDVEVKDMGVRGFTRKVGYLAKPDANLMCAQCHVEYICN

PGIDAKTGEKIGMDNRLTNHFPMVNADQIEAYYEKIGYRDFVHPLTGAKLVKMQHPDFETYLGSKHDKAGATCQNCHMPK

VKDEKTGKTYTLHWATSPRHYLKETCLSCHKDKTEAQMTSAIDAMHGYYTGKLREAESRMDEMFNAFELALAVGVDEKAL

EEARKLHSTAHVNWEYWTAVNGAWFHNPDQAVRSLAKAAKAAQDATAILRKAMAEKQKK

>OGW44789.1 hypothetical protein A2X57_05175, partial [Nitrospirae bacterium GWD2_57_8]

MQKKIIAFSVVLTAIALSFLMLSTAEAQKTAPKKKASTNNTACYGCHDTIKELHTMGKHSRVDCTYCHSGLEKHQKNPGP

DTRPATNTAWQACGQCHKEQFNSFMNVSHHRPARDEKSQLTNRAPNPFWDKLMMGHGFTKEHALTRSHNLMLIDHIIVDR

AYGGRFVPKNGWRYVLQTGKVSDVLVDREPSSSEQKKFIPQSATAANPVCLQCKTQDHILKWAYMGDPVEGAQWSRTSKV

VEVARDVQHGLNCFTCHDPHAAKPRIVRDGLIEALTRPEADTLWHKDPKRTGIKVIEMGVRGFTRKIALLDKYDSRLQCG

QCHVEYNCNPGTDPKTGEKVTMADRRTNHFPYKDVFDIYDHYVNQISFLDFRHELTGGLLWKAQHAESEVFYNSKHAKAG

VQCDNCHTPKVKDKKT
