# Supplementary material for: Structural adaptations of octaheme nitrite reductases from haloalkaliphilic Thioalkalivibrio bacteria to alkaline pH and high salinity
Source: PLoS One. 2017 May 16;12(5):e0177392. doi: 10.1371/journal.pone.0177392 (PMC5433712; doi:10.1371/journal.pone.0177392)
Supplement: S1 Fig — (DOCX) [file pone.0177392.s006.docx]

**S6. Molecular Phylogenetic analysis by Maximum Likelihood method**


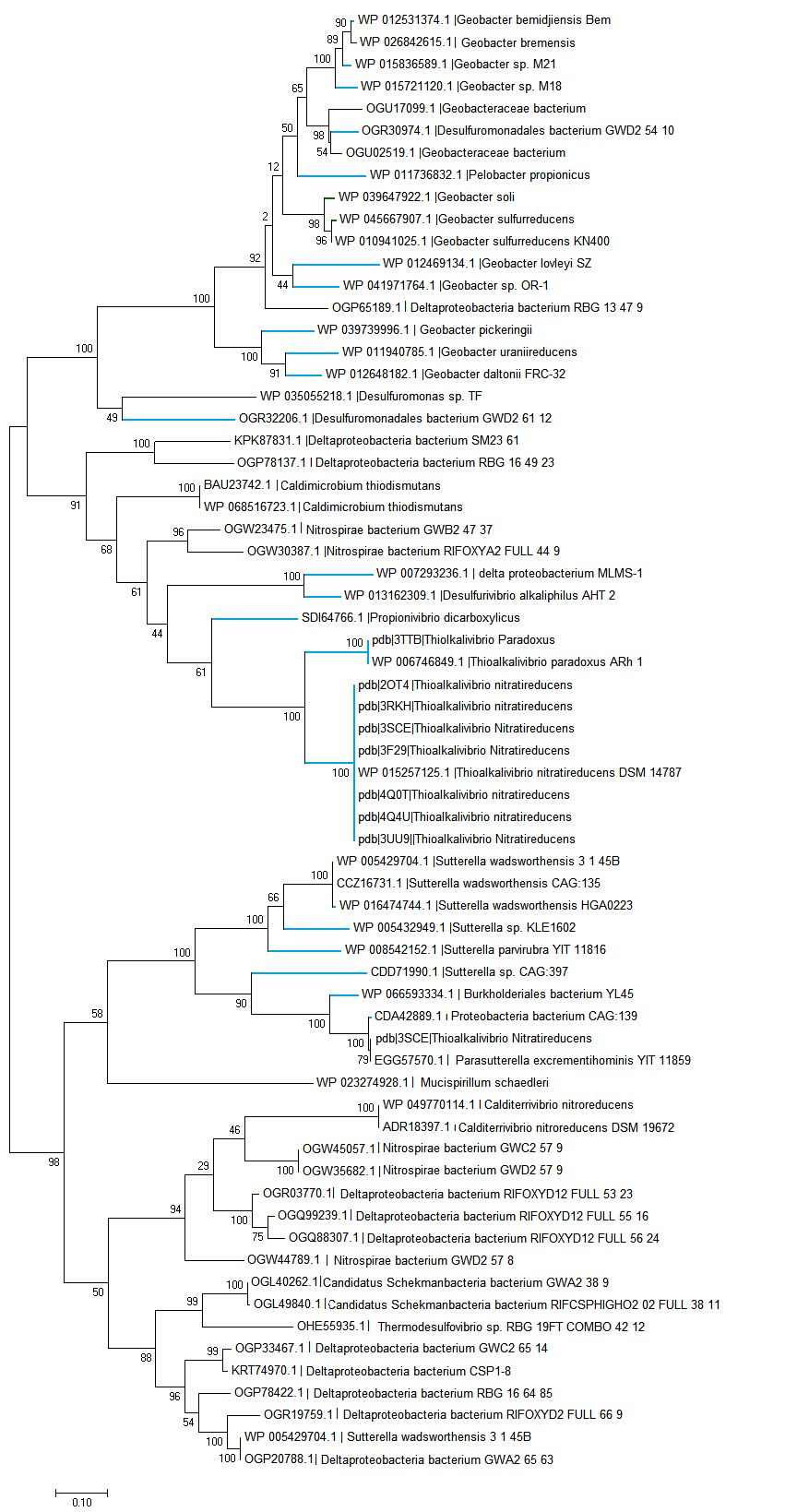


The evolutionary history was inferred by using the Maximum Likelihood method based on the JTT matrix-based model [1]. The tree with the highest log likelihood (-11017.8600) is shown. The percentage of trees in which the associated taxa clustered together is shown next to the branches. Initial tree(s) for the heuristic search were obtained automatically by applying Neighbor-Join and BioNJ algorithms to a matrix of pairwise distances estimated using a JTT model, and then selecting the topology with superior log likelihood value. The tree is drawn to scale, with branch lengths measured in the number of substitutions per site. The analysis involved 66 amino acid sequences. All positions containing gaps and missing data were eliminated. There were a total of 260 positions in the final dataset. Evolutionary analyses were conducted in MEGA7 [2]. Selected sequences are shown in green branches. Haloalkaliphiles have been highlighted with red boxes.

1. Jones D.T., Taylor W.R., and Thornton J.M. (1992). The rapid generation of mutation data matrices from protein sequences. Computer Applications in the Biosciences 8: 275-282.

2. Kumar S., Stecher G., and Tamura K. (2016). MEGA7: Molecular Evolutionary Genetics Analysis version 7.0 for bigger datasets. Molecular Biology and Evolution 33:1870-1874.
