# Supplementary material for: Structural adaptations of octaheme nitrite reductases from haloalkaliphilic Thioalkalivibrio bacteria to alkaline pH and high salinity
Source: PLoS One. 2017 May 16;12(5):e0177392. doi: 10.1371/journal.pone.0177392 (PMC5433712; doi:10.1371/journal.pone.0177392)

S11. Statistical analysis of ONSs (A - The composition of the solvent-accessible area of ONR, percentage of surface (of each residue) in the solvent-accessible area; %, B - The number of hydrophobic interactions in ONRs, The composition of hydrophobic cores of ONRs percentage of number of atoms in the core (for the residues); %

A

B
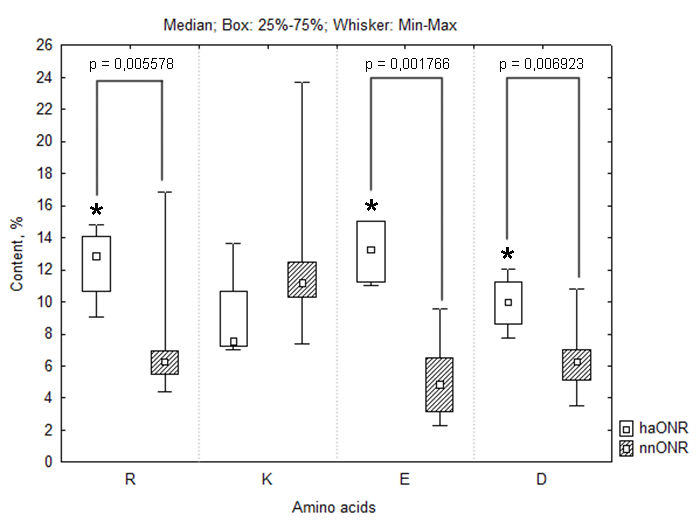


C
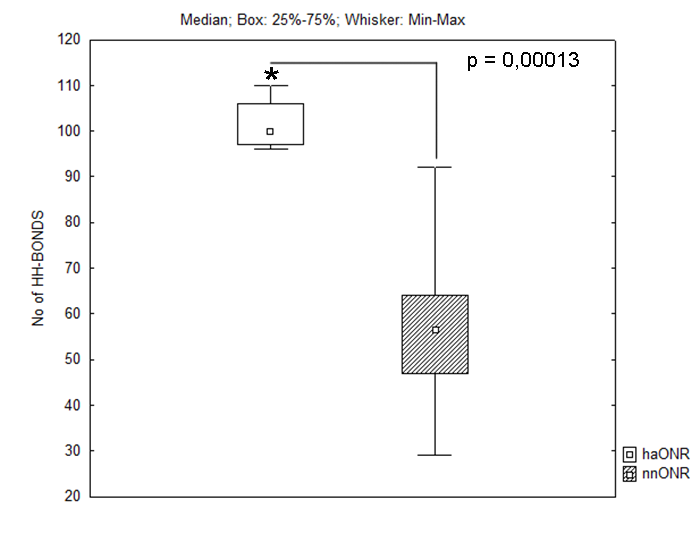


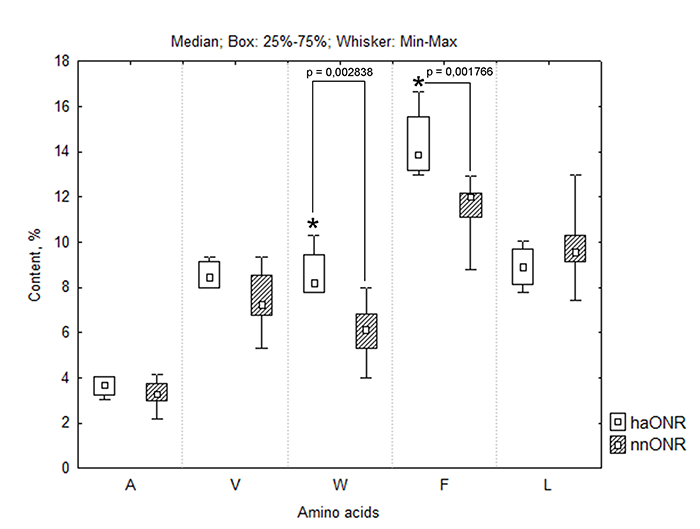

Supplement: S3 File — *—p < 0.05 for comparison with nnONR. (DOCX) [file pone.0177392.s009.docx]
